# Text Mining of Journal Articles for Sleep Disorder Terminologies

**DOI:** 10.1371/journal.pone.0156031

**Published:** 2016-05-20

**Authors:** Calvin Lam, Fu-Chih Lai, Chia-Hui Wang, Mei-Hsin Lai, Nanly Hsu, Min-Huey Chung

**Affiliations:** 1 School of Nursing, College of Nursing, Taipei Medical University, Taipei, Taiwan; 2 Department of Nursing, Taipei Medical University–Shuang Ho Hospital, New Taipei City, Taiwan; 3 Department of Nursing, Hungkuang University, Taichung, Taiwan; 4 Nursing Department, Yuanpei University, Hsinchu, Taiwan; Garvan Institute of Medical Research, AUSTRALIA

## Abstract

**Objective:**

Research on publication trends in journal articles on sleep disorders (SDs) and the associated methodologies by using text mining has been limited. The present study involved text mining for terms to determine the publication trends in sleep-related journal articles published during 2000–2013 and to identify associations between SD and methodology terms as well as conducting statistical analyses of the text mining findings.

**Methods:**

SD and methodology terms were extracted from 3,720 sleep-related journal articles in the PubMed database by using MetaMap. The extracted data set was analyzed using hierarchical cluster analyses and adjusted logistic regression models to investigate publication trends and associations between SD and methodology terms.

**Results:**

MetaMap had a text mining precision, recall, and false positive rate of 0.70, 0.77, and 11.51%, respectively. The most common SD term was *breathing-related sleep disorder*, whereas *narcolepsy* was the least common. Cluster analyses showed similar methodology clusters for each SD term, except *narcolepsy*. The logistic regression models showed an increasing prevalence of *insomnia*, *parasomnia*, and *other sleep disorders* but a decreasing prevalence of *breathing-related sleep disorder* during 2000–2013. Different SD terms were positively associated with different methodology terms regarding research design terms, measure terms, and analysis terms.

**Conclusion:**

Insomnia-, parasomnia-, and other sleep disorder-related articles showed an increasing publication trend, whereas those related to breathing-related sleep disorder showed a decreasing trend. Furthermore, experimental studies more commonly focused on hypersomnia and other SDs and less commonly on insomnia, breathing-related sleep disorder, narcolepsy, and parasomnia. Thus, text mining may facilitate the exploration of the publication trends in SDs and the associated methodologies.

## Introduction

Text mining is the process of deriving knowledge from unstructured data, such as those from text paragraphs [1–4]. To specify the direction of text mining, the necessary and unnecessary categories of studies can be assigned to automatically categorize unstructured data during the text mining process [3, 5]. Previous text mining studies have focused on topics such as methodology terms and theme extraction [4, 6, 7], publication patterns [7, 8], and effectiveness of text mining [9]. For instance, an automatic term recognition system was used for text mining and identifying methodology terms in scientific publications in the field of natural language processing; this study suggested that it is possible to text mine a particular section (e.g., Methods) of an article [7]. Peng et al. [6] identified the research themes, theorizations, and methodologies of Internet studies published during 2000–2009. The authors extracted 27,340 journal articles from Social Sciences Citation Index and Arts and Humanities Citation Index journals during the study period and used a text mining method to perform cluster analysis; the results showed that major themes in the field of Internet studies were the relationships between Internet use and specific behaviors, attitudes, or effects and the most common research methods were surveys, experiments, and content analyses. Moreover, French and Pavlidis [4] extracted neuroscience terms from the abstracts of journal articles by using the LINNAEUS text mining system to reveal the publication patterns of neuroscience articles and demonstrated that the publication of articles on brain regions and species was increasing. Ruud et al. [9] evaluated the effectiveness of text mining in detecting specific follow-up appointment criteria (i.e., date, time, physician, and appointment location) in the text of hospital discharge records. Many clinical studies have used MetaMap for text mining procedures such as identifying respiratory findings and biosurveillance in electronic emergency department reports [10], determining pneumonia and influenza deaths in electronic death records [11], and evaluating health-related quality of life in EMRs [12].

Text mining was used to facilitate research on method trends and assist researchers in obtaining associated therapies of insomnia from journal articles. Chavalarias et al. [13] performed automated text-mining analysis on 843,884 abstracts and full text of the biomedical articles during 1990–2015, which the articles were extracted from the MEDLINE and PubMed Central (PMC) databases. The results suggested that *p* values were used frequently, but other statistical information such as effect sizes and uncertainty metrics were reported less frequently in which these information should be included. Meaney et al. [14] discovered that statistical methods such as “ANOVA” and “regression” were commonly used in 113,450 published medical research articles during 1995–2015, which the articles were extracted from PMC Open-Access by using a computationally efficient text-mining approach. Besides, a previous text mining study analyzing the publication of articles on insomnia and the associated therapies [8] extracted 5,841 articles published in the past 20 years (since 1994) from the PubMed and Google Scholar databases, by performing text mining manually. The study focused on revealing the trends in studies on insomnia therapies; for instance, the number of studies on drug therapy for insomnia and its adverse effects decreased in the recent 5 years, whereas most studies examined alternative and cognitive behavioral therapies for insomnia. Text mining method was used to investigate the trends of using statistical and epidemiological methods in the journal articles.

Research identifying the disease and method associations and the publication trends in journal articles on sleep disorders (SDs) by using text mining has been limited. Moreover, statistical analyses can be performed to improve the statistical significance of text mining findings; for example, they can clarify how text-mined concepts are statistically associated. Therefore, the purpose of the current study was to apply the text mining technique to obtain information from unstructured data (journal article text) and to provide statistical support for the text mining findings by using MetaMap, cluster analyses, and logistic regression models for extracting and analyzing data from journal articles on SDs. In addition, the quality of the results obtained using MetaMap was evaluated and the possible reasons for false positives are provided. This study also used text mining to reveal the publication trends in articles on SDs and the associations between the extracted SDs and methodology terms (S1 Appendix) in journal articles published during 2000–2013.

## Materials and Methods

### Data Set

The current study conducted text mining of SD and research methodology terms in sleep-related journal articles. Journal articles were searched using the PubMed database on December 18, 2014. The keyword used for the search was *sleep*. To meet the inclusion criteria, the extracted journal articles had to be classified as journal articles, have a free full-text version available, have a publication date from January 1, 2000 to December 31, 2013, be human studies, and be written in English. Case reports and articles without abstracts were excluded. In total, 4,515 journal articles meeting the inclusion criteria were imported to Endnote X7 to search the full text in the PDF format (S2 Appendix). The PDF files were converted into text files by using Acrobat Professional DC. The titles, abstracts, and full text were extracted separately for text mining. In addition, to improve the accuracy of the text mining in indicating the study topics of the journal articles, only the methods and results sections were extracted from the full text; that is, literature review, discussion, conclusion, and references sections were excluded.

### Text Mining

Here, we used MetaMap as the text mining tool. Although MetaMap is widely used for mapping and mining clinical terms, its evaluation results for different study types remain inconsistent. For instance, Pakhomov et al. [12] identified both positive (0.76) and negative (0.78) agreement in mining EMRs for quality of life concepts; furthermore, Davis et al. [11] reported a recall of 0.998 and precision of 0.98 in mining ICD codes in records of pneumonia and influenza deaths. Chapman et al. [10] mined concepts concerning respiratory syndromes in emergency department reports and reported a recall and precision of 0.72 and 0.56, respectively. However, the authors suggested that the recall and precision values were errors in manual annotation because many uncertainties occurred during the identification of the mappings by using a standard reference, along with a problem in the domain lexicon—some concepts were not present in the Unified Medical Language System (UMLS) databases—and errors in contextual discrimination—some terms having different meanings were ambiguous during interpretation (word sense ambiguity). These evaluations may indicate that mining codes is more accurate than mining concepts. Nevertheless, MetaMap can eliminate the problems of term variants and synonymy by mapping clinical terms with terms in the UMLS database, those of negations by using the negation (NegEx) list, and those of word meaning ambiguity by using the word sense disambiguation (WSD) server [15–18]. Here, all 4,515 journal articles were imported to the Batch MetaMap 2014 server [19] for mapping the terms in the UMLS database (2015AA). To evaluate SD and methodology terms, a “start list” was created. The start list, containing the terms that were to be mined in the journal articles, was used in the MetaMap process to first map the concept unique identifier (CUI) in the UMLS database for all the SD and methodology terms. The SD terms in the start list were diagnostic terms obtained from the *Diagnostic and Statistical Manual of Mental Disorders*, fourth edition, text revision (DSM-IV-TR) and fifth edition (DSM-5) as well as the International Classification of Diseases, ninth and tenth revisions (ICD-9 and ICD-10, respectively) and their clinical modifications (ICD-9-CM and ICD-10-CM, respectively) [20]. For the start list, the methodology terms associated with research design and analysis methods were extracted from systematic reviews of nursing research [21, 22], whereas the measurement terms were extracted from systematic reviews of sleep and sleep dysfunction research [23–25]. MetaMap mapped the CUIs of all SD terms, but not all methodology terms. The unmapped terms were excluded. The final start list comprised 104 terms: 50 SD terms (mapped with 43 CUIs) in six categories and 54 methodology terms (mapped with 58 CUIs) in 11 categories (S1 Appendix). The titles, abstracts, and selected full-text sections in 4,515 journal articles were separately imported to MetaMap for mapping the terms in the UMLS database. MetaMap parsed each sentence and identified the terms. To avoid negative and ambiguous terms, the MetaMap settings were configured to process the NegEx list and WSD server and ignore the word order; other configurations were default. The results of abbreviations and negations were excluded; nevertheless, some mixed terms, such as *One-way ANOVA* and *REM Sleep Behavior Disorder*, were included (S1 Appendix). We extracted the terms by mapping the CUIs in the start list and those in the MetaMap results.

### MetaMap Evaluation

The text mining results were converted into binary data, and two codes (0 and 1) were used for each terminology variable: 0 implied that the data point (each journal article) did not contain the term, whereas 1 implied that the data point contained the term. The extracted terms in the titles, abstracts, and full text were merged (i.e., a term was coded 1 if it was present in at least one of the three sections; otherwise, it was coded 0).

We calculated the precision (also known as positive predictive value), recall (also known as true positive rate or sensitivity), and false positive rate (FPR) of the results for MetaMap. A sample of 42 journal articles (approximately 1% of 4,515 articles) was extracted using a simple random sampling method provided in SPSS for calculating the aforementioned values of MetaMap. A chi-square test was performed to determine whether the term distributions differed between the sample (n = 42) and the data set (n = 4,515 − 42 = 4,473). Two standard annotators independently manually identified the prevalence of the SD and methodology terms in the sample journal articles to determine the precision [true positives/(true positives + false positives)], recall [true positives/(true positives + false negatives)], and FPR [false positives/(false positives + true negatives) × 100%] of MetaMap. Consensus between the two references was reached before these calculations. Before evaluation, all the manually extracted terms were classified into 6 and 11 categories of SD and methodology terms, respectively. If a term—which indicated the study concept—was not found manually in a journal article but was found or indicated as the study concept by MetaMap in the same article, it was considered a false positive. Furthermore, if a term was not found manually and by MetaMap in a journal article, it was considered a true negative. In other words, only the study concept terms in the journal articles were considered true positives, and if they did not indicate the study concepts or were absent, they were considered true negatives.

### Statistical Analyses

The data set of the extracted terms in each journal article was imported to SPSS 20 for statistical analyses. Only the articles containing at least one SD term (n = 3,720) were considered for further analysis. In this paper, the word “term” refers to categories of terms (S1 Appendix). The articles were grouped into three groups on the basis of their publication years: articles published during 2000–2004, 2005–2009, and 2010–2013 were classified into Groups 1, 2, and 3, respectively. In addition, the SD and methodology terms were not mutually exclusive, implying that each data point could contain more than one SD or methodology term.

Journal articles containing at least one SD term and one methodology term were used in cluster analyses (n = 3,554), for which data were divided according to each SD term. Because the data were binary variables, average (between-groups) linkage hierarchical cluster analyses were performed using the Jaccard similarity coefficient [26, 27] to identify the similarity distance between the methodology terms for each type of SD term and to determine the terms most similar to each other in the same cluster compared with those in other clusters. Variables were matched individually to determine the Jaccard coefficient, which was 1 when both matching variables were present and 0 when both variables were absent [6, 28]. After retaining the variables having at least one matched case (i.e., the Jaccard coefficient was not 0) in each cluster analysis model, the number of clusters was determined according to the dendrogram of the model [27].

Adjusted logistic regression models were used to test for significant differences in the number of published journal articles for each SD term and the differences between methodology and SD terms. In adjusted logistic regression models, each SD term was included as a dependent variable, whereas each methodology term and year group were included as independent variables. Group 1 and the code 0 (absence of the term) for all SD and methodology terms were the reference categories.

## Results

The chi-square test results showed no significant differences in term distributions between the sample and the data set (p = 0.488). The sample journal articles had 714 mappings, and the results of the manual identification showed that the precision, recall, and FPR were 0.70, 0.77, and 11.51%, respectively; inter-rater reliability (kappa: 0.92, 95% CI 0.88–0.95) indicated high consistency for the two references. Table 1 shows the positives and negatives of MetaMap mappings and five possible reasons for false positives: 18.03% of false positives were attributable to related work, 6.56% were attributable to domain lexicon, 11.48% were attributable to negation error, 4.92% were attributable to complicated sentence structures, and 59.02% were attributable to semantic inference.

**Table 1:** Positives and negatives of MetaMap mappings in 42 sample journal articles (n = 714).

Classification	n (%)
True positives	142 (19.89)
True negatives	469 (65.69)
False negatives	42 (5.88)
False positives	61 (8.54)
Related work	11 (18.03)
Domain lexicon	4 (6.56)
Negation error	7 (11.48)
Complicated sentence structure	3 (4.92)
Semantic inference	36 (59.02)

MetaMap identified that 3,720 of 4,515 journal articles contained SD and methodology terms. Table 2 shows the number (n) of journal articles containing the extracted SD and research methodology terms. The number of journal articles containing each term is stated in S1 Appendix. The most common SD term was *breathing-related sleep disorder* (n = 2,351), whereas the least common was *narcolepsy* (n = 209).

**Table 2:** Distribution of SD and methodology terms (number of journal articles) from 2000 to 2013 (n = 3,720).

Term	n (%)
**SD**	
*Insomnia*	447 (12.02)
*Breathing-related sleep disorder*	2,351 (63.20)
*Hypersomnia*	567 (15.24)
*Narcolepsy*	209 (5.62)
*Parasomnia*	962 (25.86)
*Other sleep disorders*	2,110 (56.72)
**Methodology**	
***Design***	
*Observational study*	492 (13.23)
*Correlational study*	728 (19.57)
*Experimental study*	446 (11.99)
*Meta-analysis*	28 (0.75)
***Measurement***	
*Objective measure*	2,627 (70.62)
*Subjective measure*	1,138 (30.59)
***Analysis***	
*Reliability and validity*	
*Reliability*	873 (23.47)
*Validity*	412 (11.08)
*Descriptive statistics*	2,278 (61.24)
*Parametric test*	2,900 (77.96)
*Nonparametric test*	499 (13.41)

Table 3 shows the results of the hierarchical cluster analyses, which identified clusters of methodology terms in all journal articles containing at least one of the seven SD terms. The results showed that the clusters for most SDs were similar: in all groups, Cluster 1 contained *observational study*, *correlational study*, *experimental study*, *objective measure*, *subjective measure*, *reliability*, *validity*, *descriptive statistics*, *parametric test*, and *nonparametric test*, whereas Cluster 2 contained only *meta-analysis*. However, in the *narcolepsy* group, Cluster 1 contained *observational study*, *correlational study*, *objective measure*, *subjective measure*, *reliability*, *validity*, *descriptive statistics*, *parametric test*, and *nonparametric test*, whereas Cluster 2 contained only *experimental study*.

**Table 3:** Average linkage hierarchical cluster analyses of research methodology terms used in relation to each SD term in the journal articles from 2000 to 2013.

SD n (%)^†^	Cluster^‡^	Methodology terms
All SDs	Cluster 1	*Observational study*, *Correlational study*, *Experimental study*, *Objective measure*, *Subjective measure*, *Reliability*, *Validity*, *Descriptive statistics*, *Parametric test*, *Nonparametric test*
3,670 (100)		
	Cluster 2	*Meta-analysis*
*Insomnia*	Cluster 1	*Observational study*, *Correlational study*, *Experimental study*, *Objective measure*, *Subjective measure*, *Reliability*, *Validity*, *Descriptive statistics*, *Parametric test*, *Nonparametric test*
435 (11.85)		
	Cluster 2	*Meta-analysis*
*Breathing- related sleep disorder*	Cluster 1	*Observational study*, *Correlational study*, *Experimental study*, *Objective measure*, *Subjective measure*, *Reliability*, *Validity*, *Descriptive statistics*, *Parametric test*, *Nonparametric test*
2,334 (63.60)	Cluster 2	*Meta-analysis*
*Hypersomnia*	Cluster 1	*Observational study*, *Correlational study*, *Experimental study*, *Objective measure*, *Subjective measure*, *Reliability*, *Validity*, *Descriptive statistics*, *Parametric test*, *Nonparametric test*
560 (15.26)		
	Cluster 2	*Meta-analysis*
*Narcolepsy*	Cluster 1	*Observational study*, *Correlational study*, *Objective measure*, *Subjective measure*, *Reliability*, *Validity*, *Descriptive statistics*, *Parametric test*, *Nonparametric test*
208 (5.67)		
	Cluster 2	*Experimental study*
*Parasomnia*	Cluster 1	*Observational study*, *Correlational study*, *Experimental study*, *Objective measure*, *Subjective measure*, *Reliability*, *Validity*, *Descriptive statistics*, *Parametric test*, *Nonparametric test*
946 (25.78)		
	Cluster 2	*Meta-analysis*
*Other sleep disorders*	Cluster 1	*Observational study*, *Correlational study*, *Experimental study*, *Objective measure*, *Subjective measure*, *Reliability*, *Validity*, *Descriptive statistics*, *Parametric test*, *Nonparametric test*
2,090 (56.95)		
	Cluster 2	*Meta-analysis*

^†^n (%): Only articles containing at least one SD term and one methodology term for each type of SD were used.

^‡^Clusters: methodology terms were retained in each cluster analysis of SD when at least one match of the methodology terms was observed.

**Fig 1:**
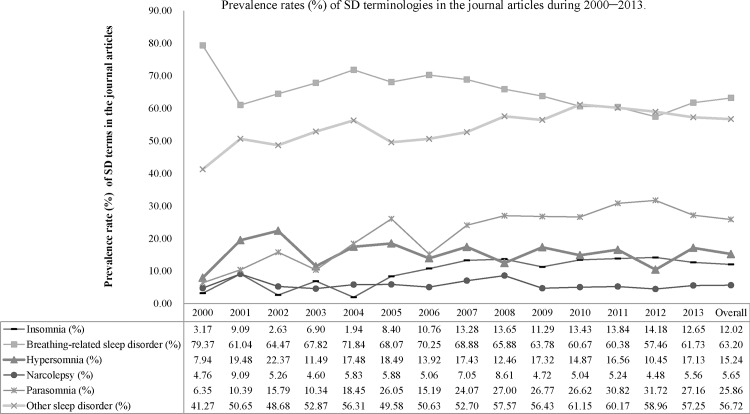
Prevalence rate of sleep disorder (SD) terms in the journal articles during 2000–2013.

Fig 1 shows the trends of the prevalence of SD terms in the journal articles from 2000 to 2013. The prevalence rates of *insomnia*, *parasomnia*, and *other sleep disorder* increased, whereas those of *breathing-related sleep disorder* decreased and those of *hypersomnia* and *narcolepsy* remained unchanged. Table 4 shows the results of adjusted logistic regression models for the association between methodology terms and each SD term in the journal articles. The models showed that compared with the 2000–2004 period, publications containing *insomnia* (adjusted odds ratio (AOR): 2.48, 95% confidence interval (CI) 1.51–4.09) and *parasomnia* (AOR: 2.08, 95% CI 1.49–2.91) increased during the 2005–2009 and 2010–2013 periods (AOR: 2.58, 95% CI 1.58–4.19 and AOR: 2.40, 95% CI 1.74–3.31, respectively); publications containing *other sleep disorder* (AOR: 1.26, 95% CI 1.01–1.57) increased during the 2010–2013 period; and publications containing *breathing-related sleep disorder* (AOR: 0.65, 95% CI 0.50–0.84) decreased during the 2010–2013 period.

**Table 4:** Adjusted logistic regression models for SD and research methodology terms in the journal articles from 2000 to 2013.

Variable	*Insomnia*	*Breathing- related sleep disorder*	*Hypersomnia*	*Narcolepsy*	*Parasomnia*	*Other sleep disorders*
	AOR^†^	AOR	AOR	AOR	AOR	AOR
	(95% CI)	(95% CI)	(95% CI)	(95% CI)	(95% CI)	(95% CI)
	n = 447	n = 2,351	n = 567	n = 209	n = 962	n = 2,110
**Year**						
2000–2004	Ref	Ref	Ref	Ref	Ref	Ref
2005–2009	2.48	0.86	0.97	0.94	2.08	1.10
	(1.51–4.09)**	(0.66–1.13)	(0.71–1.32)	(0.58–1.52)	(1.49–2.91)**	(0.87–1.39)
2010–2013	2.58	0.65	0.89	0.72	2.40	1.26
	(1.58–4.19)**	(0.50–0.84)**	(0.66–1.20)	(0.45–1.15)	(1.74–3.31)**	(1.01–1.57)*
**Methodology**^‡^						
***Design***						
*Observational study*	1.11	1.84	0.96	1.26	0.96	0.86
	(0.83–1.49)	(1.44–2.34)**	(0.73–1.26)	(0.85–1.85)	(0.76–1.21)	(0.71–1.05)
*Correlational study*	1.19	0.84	0.87	0.90	1.22	1.11
	(0.93–1.52)	(0.70–1.02)	(0.69–1.10)	(0.63–1.30)	(1.01–1.48)*	(0.93–1.31)
*Experimental study*	0.64	0.39	1.36	0.50	0.42	1.72
	(0.44–0.93)*	(0.31–0.49)**	(1.05–1.76)*	(0.28–0.91)*	(0.31–0.56)**	(1.39–2.13)**
*Meta-analysis*	1.41	1.02	0.47	0	0.34	1.02
	(0.47–4.23)	(0.44–2.39)	(0.11–2.00)		(0.10–1.19)	(0.48–2.19)
***Measurement***						
*Objective measure*	0.47	4.65	0.97	2.22	0.37	0.63
	(0.38–0.58)**	(3.97–5.45)**	(0.79–1.18)	(1.51–3.27)**	(0.31–0.43)**	(0.54–0.74)**
*Subjective measure*	1.46	1.46	1.27	2.24	1.31	1.35
	(1.18–1.81)**	(1.23–1.72)**	(1.05–1.54)*	(1.68–2.99)**	(1.10–1.55)*	(1.16–1.57)**
***Analysis***						
*Reliability*	1.80	0.56	1.02	0.99	2.02	1.64
	(1.41–2.29)**	(0.47–0.68)**	(0.81–1.30)	(0.68–1.44)	(1.66–2.44)**	(1.37–1.97)**
*Validity*	1.58	0.56	1.05	1.60	2.48	1.54
	(1.17–2.12)*	(0.43–0.72)**	(0.76–1.44)	(1.01–2.55)*	(1.94–3.18)**	(1.19–1.99)**
*Descriptive statistics*	0.86	1.60	1.03	1.00	0.78	0.86
	(0.70–1.06)	(1.38–1.87)**	(0.86–1.25)	(0.75–1.35)	(0.66–0.92)*	(0.75–0.99)*
*Parametric test*	1.19	0.86	1.10	0.73	1.05	1.25
	(0.92–1.55)	(0.71–1.03)	(0.88–1.37)	(0.52–1.01)	(0.86–1.28)	(1.07–1.47)*
*Nonparametric test*	0.75	1.36	0.76	0.97	0.97	0.95
	(0.53–1.05)	(1.07–1.72)*	(0.57–1.01)	(0.65–1.46)	(0.76–1.23)	(0.78–1.15)

Note:

*p < 0.05

**p ≤ 0.001.

^†^AOR: adjusted odds ratio.

Zero (0) odds ratio indicates no match occurred; other numbers for odds ratio indicate at least one match occurred.

^‡^Reference group: not containing that methodology term.

For research design terms, *experimental study* was more common in the publications containing *hypersomnia* (AOR: 1.36, 95% CI 1.05–1.76) or *other sleep disorder* (AOR: 1.72, 95% CI 1.39–2.13). *Observational study* was more common in the publications containing *breathing-related sleep disorder* (AOR: 1.84, 95% CI 1.44–2.34). *Correlational study* was more common in the publications containing *parasomnia* (AOR: 1.22, 95% CI 1.01–1.48).

For measurement terms, *subjective measure* was more common in the publications containing one of the six SD terms (*insomnia*, AOR: 1.46, 95% CI 1.18–1.81; *breathing-related sleep disorder*, AOR: 1.46, 95% CI 1.23–1.72; *hypersomnia*, AOR: 1.27, 95% CI 1.05–1.54; *narcolepsy*, AOR: 2.24, 95% CI 1.68–2.99; *parasomnia*, AOR: 1.31, 95% CI 1.10–1.55; or *other sleep disorder*, AOR: 1.35, 95% CI 1.16–1.57). *Objective measure* was more common in publications containing *breathing-related sleep disorder* (AOR: 4.65, 95% CI 3.97–5.45) or *narcolepsy* (AOR: 2.22, 95% CI 1.51–3.27).

For analysis terms, *reliability* and *validity* were more common in the publications containing *insomnia* (AOR: 1.80, 95% CI 1.41–2.29 and AOR: 1.58, 95% CI 1.17–2.12; respectively), *parasomnia* (AOR: 2.02, 95% CI 1.66–2.44 and AOR: 2.48, 95% CI 1.94–3.18; respectively), or *other sleep disorder* (AOR: 1.64, 95% CI 1.37–1.97 and AOR: 1.54, 95% CI 1.19–1.99; respectively). By contrast, *reliability* (AOR: 0.56, 95% CI 0.47–0.68) and *validity* (AOR: 0.56, 95% CI 0.43–0.72) were less common in the publications containing *breathing-related sleep disorder*, but *descriptive statistics* (AOR: 1.60, 95% CI 1.38–1.87) and *nonparametric test* (AOR: 1.36, 95% CI 1.07–1.72) were more common. *Parametric test* was more common in the publications containing *other sleep disorder* (AOR: 1.25, 95% CI 1.07–1.47).

## Discussion

This study is the first to use text mining data from MetaMap for investigating the publication trends of articles containing SD terms and the associations of these terms with research methodology terms. By evaluating the precision and recall of MetaMap, we validated the quality of the text-mined SD and methodology terms used in journal articles. We used the following MetaMap settings to improve the precision and recall: NegEX list; WSD server; ignoring word order; and processing of only the title, abstract, and methods and results sections. In addition, an 11.51% FPR indicated an acceptable quality of the current results, and false positives were classified into five error categories, which may serve as references for further investigation on the validity of MetaMap.

### MetaMap Evaluation

Our MetaMap evaluation showed a precision (0.70) and recall (0.77) that were lower than those reported by Davis et al. (precision, 0.98; recall, 0.998) [11], probably because Davis et al. mined ICD codes rather than word concepts in their data, which may have resulted in less ambiguous mappings. By contrast, our precision and recall were higher than those reported by Chapman et al. (precision, 0.56; recall, 0.72) [10], regardless of current mapping criterion is the studied concepts (for positives) or concepts that were not studied (for negatives) in the journal articles. The possible reasons for this may be that this study included the title, abstract, and methods and results sections of the full text and eliminated the problems of negations and word sense ambiguity, which may reduce the false mappings in the results. Moreover, the standard reference in the present study was clear; there was no uncertainty regarding whether the terms in the journal articles were SD or research methodology terms during the manual mappings because the mappings were limited to the selected ICD and *DSM* concepts.

Although our MetaMap mappings considered negation and word sense ambiguity, the FPR was 11.51%, indicating that 11.51% of all negatives were falsely identified as positives. Table 1 shows the false positives classified into five error categories: related work, domain lexicon, negation, complicated sentence structure, and semantic inference. Some false positives were obtained because of the related work cited the Methods sections; for example, the reliability of a used scale was cited from another study. Some errors were observed in the domain lexicon because some of the concepts such as the names of institutions (e.g., American Sleep Disorders Association) and measurements (e.g., Structured Insomnia Interview) are not provided in the UMLS database. However, the words inside these phrases (e.g., SDs and insomnia), which did not indicate the actual study concepts of the journal articles, were identified by MetaMap. Some errors occurred because the negation function of MetaMap failed to identify the negation terms, such as “free of,” or “ruled out.” Furthermore, some sentence structures were highly complicated and limited the negation function of MetaMap; for example, in the sentence “patients are excluded if they had been referred for reasons other than the evaluation of suspected sleep-disordered breathing (e.g., narcolepsy or movement disorder),” the term “narcolepsy” should be a negated term. However, the word “excluded” was not near the word “narcolepsy,” making it difficult for MetaMap to identify whether it was a negated term. The most severe error (59.02%) was that in semantic inference, where MetaMap overly accepted some relevant SD terms; for instance, “augmented sleep” was identified as “hypersomnia” and “sleep problem” as “dyssomnia” or “parasomnia,” although these terms did not represent the actual diagnoses of SD in the ICD or *DSM*.

### Publication Trends

This study revealed the publication trends in journal articles on SDs and the associated research methodologies through text mining. Fig 1 and Table 4 show that the prevalence of all SD terms in the journal articles regarding insomnia, parasomnia, and other SDs increased, indicating increased attention; by contrast, studies on breathing-related SD decreased, indicating decreased attention. Furthermore, during the entire study period, the most studied topic was breathing-related SD, whereas the least studied was narcolepsy.

### SD and Methodology Terms

Hierarchical cluster analyses (Table 3) showed similar patterns for methodology clusters in SD terms, indicating that the methodology terms were similar in each SD, except *narcolepsy* (no association between *meta-analysis* and *narcolepsy*). Table 2 shows that the least common term was *narcolepsy*. Thus, a meta-analysis of narcolepsy is warranted for further exploration in the future. *Experimental study* was more commonly associated with *hypersomnia* and *other sleep disorder*; the articles tended to concern the effects of interventions, such as the effects of sleep restriction, medicine, light therapy, or hormones, and they used *subjective measures* (scales or questionnaires) for identifying the subjects for the studies [29, 30]. *Reliability* and *validity* were more common in the *experimental study* articles. In addition, the studies regarding other sleep disorder tended to use parametric tests to show the results because they had an adequately large sample size. Furthermore, the more common research designs for breathing-related sleep disorder and parasomnia studies were observational and correlational study, respectively. Studies on breathing-related sleep disorder more commonly used objective and subjective measures to investigate the risk factors. In addition, our results showed a higher prevalence (AOR: 4.65) of the use of objective measures; consequently, reliability and validity were less commonly used for validation. Nevertheless, these studies more commonly used nonparametric tests because their sample sizes were usually <30 [31–35]. The studies on parasomnia used a subjective measure (such as questionnaires) more commonly than an objective measure to investigate the prevalence and risk factors [36–38]. Moreover, reliability and validity were more commonly evaluated in studies using a subjective measure.

### Limitations

This study had some limitations that should be considered when interpreting the findings. The PubMed database may not have contained all journal articles on SDs published during the study period; this may have affected the current results, such as those regarding publication trends. In addition, the current list of terms was extracted from the *DSM* and ICD, which may not include all SD and methodology terms. Moreover, MetaMap highly relies on the UMLS database, which does not contain some methodology terms; consequently, these terms could not be analyzed. These limitations may have led to underestimation of the number of journal articles for each term. Moreover, specific sections of journal articles, namely the title, abstract, and methods and results sections, were text subjected to text mining to obtain more accurate mining result; thus, terms from the literature review and discussion sections may have been excluded. Nevertheless, the current study assumed that the titles, abstracts, and full text precisely represent the research themes of the studies. The current findings may have been underestimated because the investigated SD and methodology terms may have been stated unclearly. Furthermore, this study used terms in the journal articles to identify the studied SD and used research methodologies. To evaluate the identification results, we also text mined 1% of the journal articles manually to calculate the precision and recall of MetaMap and provided the possible reasons for false positive identification, thus providing evidence on the data quality and insights into future improvements of MetaMap. In addition, SD and methodology terms in each journal article were not mutually exclusive, implying that a journal article could contain more than one type of SD or methodology term. For instance, a journal article could contain both *insomnia* and *hypersomnia*; in this case, the journal article could not be classified into only one of these two categories. Consequently, comparisons among different SDs and methodologies were not performed.

## Conclusion

The precision and recall of MetaMap for text mining SD and the associated research methodology terms improved. Regarding the publication trends, the most common SD term during the study period of 2000–2013 was *breathing-related sleep disorder*, whereas *narcolepsy* was the least common. Most methodology terms were in the same cluster for each group of SD terms, except *narcolepsy*. Insomnia-, parasomnia-, and other sleep disorder-related articles showed increasing publication trends, whereas those related to breathing-related sleep disorder showed decreasing publication trends during 2000–2013. Hypersomnia and other sleep disorder were more common in experimental studies using a subjective measure to identify subjects for the articles. Moreover, other sleep disorder-related studies (having adequately large sample sizes) more commonly used a parametric test. The common designs for breathing-related sleep disorder and parasomnia studies were observational and correlational, respectively, and both used subjective measures. Studies containing *breathing-related sleep disorder* had a higher prevalence of *objective measure* but a lower prevalence of *reliability*, *validity*, and *nonparametric test*. Text mining by using MetaMap may facilitate identifying the corresponding terms to indicate the publication trends of the studied SD and used research methodologies in journal articles.

## Supporting Information

S1 Appendix List of SD and research methodology terms from 2000 to 2013 (n = 3,720). (DOCX)

S2 Appendix List of PMIDs for the studied journal articles (n = 4,515). (DOCX)
