# Supplementary material for: Text Mining of Journal Articles for Sleep Disorder Terminologies
Source: PLoS One. 2016 May 20;11(5):e0156031. doi: 10.1371/journal.pone.0156031 (PMC4874549; doi:10.1371/journal.pone.0156031)
Supplement: S1 Appendix — (DOCX) [file pone.0156031.s001.docx]

**S1 Appendix**. List of SD and research methodology terms from 2000 to 2013 (n = 3,720)

| Term | UMLS Term | CUI | No.^†^ |
| --- | --- | --- | --- |
| **Sleep Disorder** |  |  |  |
| ***Insomnia*** | Sleeplessness | C0917801 | 447 |
| *Nonorganic Insomnia* | Nonorganic Insomnia | C0349255 | 2 |
| *Primary Insomnia* | Primary Insomnia | C0033139 | 103 |
| *Insomnia due to medical condition* | Insomnia due to Medical Conditions | C2875252 | 0 |
| *Hypersomnia due to medical condition* | Hypersomnia due to Medical Conditions | C2875253 | 0 |
| *Organic sleep disorder* | Organic sleep disorder | C1561892 | 2 |
| ***Breathing-related sleep disorder*** | Breathing-related Sleep Disorder | C0236845 | 2,351 |
| *Obstructive sleep apnea* | Sleep Apnea, Obstructive | C0520679 | 1,600 |
| *Central sleep apnea* | Sleep Apnea, Central | C0520680 | 373 |
| *Cheyne Stokes breathing / Cheyne Stokes respiration* | Cheyne–Stokes Respiration | C0008039 | 44 |
| *Periodic breathing* | Respiration intermittent | C1313952 | 79 |
| *Sleep apnea* | Sleep Apnea Syndromes | C0037315 | 1,557 |
| *Sleep related hypoventilation* | Sleep Hypoventilation | C2732337 | 6 |
| *Idiopathic sleep related nonobstructive alveolar hypoventilation* | Idiopathic Sleep-related Non-obstructive Alveolar Hypoventilation | C2711232 | 0 |
| *Congenital central alveolar hypoventilation syndrome* | Congenital Central Hypoventilation | C1275808 | 3 |
| *Circadian rhythm sleep disorder* | Sleep Disorders, Circadian Rhythm | C0877792 | 21 |
| *Circadian rhythm sleep disorder of nonorganic origin* | Circadian Rhythm Sleep Disorder of Nonorganic Origin | C1561844 | 0 |
| *Delayed sleep phase type* | Delayed Sleep Phase | C0854740 | 19 |
| *Advanced sleep phase type* | Advanced Sleep Phase | C0854739 | 5 |
| *Irregular sleep wake type* | Circadian Rhythm Sleep Disorder, Irregular Sleep-Wake Type | C1561874 | 0 |
| *Shift work type* | Work-Shift Change (Disorder) | C0393773 | 0 |
| *Jet lag type* | Jet Lag Syndrome | C0231311 | 14 |
| *Free running type* | Non-24 hour Sleep–Wake Cycle | C0393772 | 0 |
| ***Hypersomnia*** | Hypersomnia | C0917799 | 567 |
| *Hypersomnolence disorder* | Disorders of Excessive Somnolence | C0020524 | 14 |
| *Hypersomnolence* | Hypersomnolence | C0751229 | 26 |
| *Primary hypersomnia* | Primary Hypersomnia | C0033138 | 0 |
| *Other organic hypersomnia* | Hypersomnia Disorder Related to a Known Organic Factor | C0270543 | 0 |
| *Nonorganic hypersomnia /*  *Hypersomnia of non-organic origin* | Hypersomnia of Non-organic Origin | C1305395 | 0 |
| ***Narcolepsy*** | Narcolepsy | C0027404 | 209 |
| *Narcolepsy cataplexy syndrome* | Narcolepsy–Cataplexy Syndrome | C0751362 | 27 |
| *Narcolepsy without cataplexy* | Narcolepsy without Cataplexy | C1456240 | 2 |
| ***Parasomnia*** | Parasomnia | C0030508 | 962 |
| *Sleep arousal disorder* | Sleep Arousal Disorders | C0752294 | 9 |
| *Somnambulism / sleepwalking* | Somnambulism | C0037672 | 54 |
| *Sleep terror / night terror* | Night Terrors | C0037320 | 31 |
| *Nightmare disorder* | Nightmares | C0028084 | 113 |
| *Dyssomnia* | Dyssomnias | C0700201 | 633 |
| *Rapid eye movement sleep behavior disorder* | REM Sleep Behavior Disorder | C0751772 | 105 |
| *Restless leg syndrome* | Restless Legs Syndrome | C0035258 | 230 |
| ***Other sleep disorder*** |  |  | 2,110 |
| *Organic insomnia* | Insomnia Disorder-related to Known Organic Factor | C0021607 | 3 |
| *Other sleep disturbance* | Sleep Disturbances | C0037317 | 1105 |
| *sleep disorder unspecified / other specified sleep wake disorder* | Sleep Disorders | C0851578 | 1407 |
|  |  |  |  |
| **Methodology** |  |  |  |
| ***Design*** |  |  |  |
| ***Observational study*** |  |  | 492 |
| *Case control* | Case–Control Studies | C0007328 | 75 |
| *Cohort study* | Cohort Studies | C0009247 | 250 |
| *Retrospective research* | Retrospective Studies | C0035363 | 188 |
| ***Correlational study*** |  |  | 728 |
| *Correlational research* | Correlation Study | C0010101 | 247 |
| *Longitudinal research* | Longitudinal Studies | C0023981 | 504 |
| *Survey* | Survey Research | C0683942 | 8 |
| ***Experimental study*** |  |  | 446 |
| *Experimental research* | Research Study | C0681814 | 440 |
| *Quasi experimental research design* | Quasi-Experimental Research Design / Quasi Experimental Methods / Non-randomized Controlled Trials as Topic | C0815255/C1510570/C2985410 | 6 |
| ***Meta-analysis*** | Meta-analysis | C0920317 | 28 |
|  |  |  |  |
| ***Measurement*** |  |  |  |
| ***Objective Measure*** |  |  | 2,627 |
| *Actigraphy* | Actigraphy | C1171301 | 508 |
| *Bed occupancy sensor* | Bed/Chair Occupancy Alarm Sensor | C3877535 | 0 |
| *Multiple sleep latency test* | Multiple Sleep Latency Test | C0519186 | 111 |
| *Polysomnography* | Polysomnography | C0162701 | 2459 |
| ***Subjective Measure*** |  |  | 1,138 |
| *Epworth sleepiness scale* | Epworth Sleepiness Scale Questionnaire | C3541276 | 791 |
| *Fatigue severity scale* | Fatigue Severity Scale | C3813323 | 22 |
| *Multidimensional fatigue inventory* | Multidimensional Fatigue Inventory | C1881923 | 15 |
| *Pittsburg sleep quality index* | Pittsburg Sleep Quality Index / Pittsburgh Sleep Quality Index | C3641648/C3697468 | 452 |
|  |  |  |  |
| ***Analysis*** |  |  |  |
| ***Reliability and validity*** |  |  |  |
| ***Reliability*** | Reliability | C2347947 | 873 |
| *Test retest reliability* | Test–Retest Reliability | C0237828 | 111 |
| *Internal consistency* | Internal Consistency | C0870731 | 241 |
| *Interrater reliability* | Interrater Reliability | C0870740 | 93 |
| *Kappa* | Kappa | C0439099 | 84 |
| ***Validity*** | Validity | C2349101 | 412 |
| *Concurrent validity* | Concurrent Validity | C0871697 | 29 |
| *Construct validity* | Construct Validity | C0681897 | 69 |
| *Content validity* | Content Validity | C1510592 | 14 |
| *Criterion related validity* | Criterion Validity | C2699472 | 19 |
| *Predictive validity* | Predictive Validity | C0681898 | 13 |
| ***Descriptive statistics*** |  |  | 2,278 |
| *Mean* | Population Mean | C2347634 | 1,609 |
| *Median* | Median Statistical Measurement | C0876920 | 401 |
| *Standard deviation* | Standard Deviation | C0871420 | 991 |
| ***Parametric test*** |  |  | 2,900 |
| *Chi square* | Chi-square Test | C1552646 | 238 |
| *t test* | *t* Test | C0871472 | 272 |
| *Paired samples t test* | Paired *t* Test | C1709451 | 432 |
| *Analysis of variance* | Analysis of Variance | C0002780 | 597 |
| *One way analysis of variance* | One-way Analysis of Variance | C1709320 | 273 |
| *Two way analysis of variance* | Two-way Analysis of Variance | C1710497 | 73 |
| *Analysis of covariance* | ANCOVA | C0814908 | 159 |
| *Multivariate analysis of the variance* | Multiple Analysis of Variance | C0681926 | 4 |
| *Correlation* | Correlation | C1707520 | 1729 |
| *Regression* | Regression Analysis | C0034980 | 587 |
| *Linear regression* | Linear Regression | C0023733 | 523 |
| *Logistic regression* | Logistic Regression | C0206031 | 504 |
| *Poisson regression* | Poisson Distribution | C0032347 | 5 |
| *Factor analysis* | Analysis, Factor | C0085801 | 90 |
| *Confirmatory factor analysis* | Confirmatory Factor Analysis | C0870334 | 16 |
| *Survival analysis* | Survival Analysis | C0038953 | 29 |
| *Item analysis* | Statistical Item Analysis / Test Item Analysis | C0237692/C0237693 | 1 |
| *Path analysis* | Path Analysis | C0683963 | 8 |
| *Structural equation modeling* | Structural Equation Modeling | C0681947 | 28 |
| ***Nonparametric Test*** |  |  | 499 |
| *Kruskal Wallis test* | Kruskal–Wallis Test | C1708614 | 79 |
| *Mann Whitney U test* | Mann–Whitney U Test | C0242927 | 177 |
| *Mann Whitney test* | Mann–Whitney Test | C1708930 | 88 |
| *Spearman rank correlation* | Spearman Correlation Test | C1710141 | 248 |

^†^No.: Number of journal articles.
