# Supplementary material for: Text Mining of Journal Articles for Sleep Disorder Terminologies
Source: PLoS One. 2016 May 20;11(5):e0156031. doi: 10.1371/journal.pone.0156031 (PMC4874549; doi:10.1371/journal.pone.0156031)
Supplement: S2 Appendix — (DOCX) [file pone.0156031.s002.docx]

**S2 Appendix**. List of PMIDs for the studied journal articles (n = 4,515)

| 10653830 |
| --- |
| 10679542 |
| 10700663 |
| 10702169 |
| 10705268 |
| 10706499 |
| 10706500 |
| 10710392 |
| 10712296 |
| 10714417 |
| 10722770 |
| 10722771 |
| 10733682 |
| 10733684 |
| 10733685 |
| 10733686 |
| 10733690 |
| 10749783 |
| 10759455 |
| 10759458 |
| 10770821 |
| 10770822 |
| 10778031 |
| 10778032 |
| 10780757 |
| 10780758 |
| 10781097 |
| 10797148 |
| 10805822 |
| 10818083 |
| 10849239 |
| 10849241 |
| 10849242 |
| 10849243 |
| 10849244 |
| 10849246 |
| 10867594 |
| 10869008 |
| 10878111 |
| 10885414 |
| 10899061 |
| 10904043 |
| 10904052 |
| 10926630 |
| 10933096 |
| 10933098 |
| 10940788 |
| 10950890 |
| 10950891 |
| 10956244 |
| 10979246 |
| 10984335 |
| 11001083 |
| 11007559 |
| 11012862 |
| 11012863 |
| 11012866 |
| 11012867 |
| 11012868 |
| 11012869 |
| 11012870 |
| 11012871 |
| 11012872 |
| 11040153 |
| 11050263 |
| 11070456 |
| 11070457 |
| 11080060 |
| 11083891 |
| 11085964 |
| 11090602 |
| 11106205 |
| 11106206 |
| 11106207 |
| 11106208 |
| 11123213 |
| 11123521 |
| 11123523 |
| 11136690 |
| 11153591 |
| 11153593 |
| 11158285 |
| 11159301 |
| 11160467 |
| 11163282 |
| 11163547 |
| 11181609 |
| 11181622 |
| 11212164 |
| 11223727 |
| 11247947 |
| 11274257 |
| 11276566 |
| 11285050 |
| 11285051 |
| 11285052 |
| 11285053 |
| 11285054 |
| 11285056 |
| 11285057 |
| 11287828 |
| 11306557 |
| 11309469 |
| 11331215 |
| 11353661 |
| 11356653 |
| 11386202 |
| 11386203 |
| 11386204 |
| 11388497 |
| 11392581 |
| 11392586 |
| 11404230 |
| 11405526 |
| 11408436 |
| 11408458 |
| 11413347 |
| 11413348 |
| 11414343 |
| 11416243 |
| 11416244 |
| 11416253 |
| 11422722 |
| 11422729 |
| 11422730 |
| 11434366 |
| 11440916 |
| 11464081 |
| 11488314 |
| 11488315 |
| 11491173 |
| 11491174 |
| 11503285 |
| 11509518 |
| 11510789 |
| 11510790 |
| 11511706 |
| 11511716 |
| 11511794 |
| 11514695 |
| 11529293 |
| 11529294 |
| 11530895 |
| 11534879 |
| 11546826 |
| 11562521 |
| 11576363 |
| 11580006 |
| 11589351 |
| 11589352 |
| 11641138 |
| 11682272 |
| 11682274 |
| 11682275 |
| 11682276 |
| 11682279 |
| 11694810 |
| 11696069 |
| 11696070 |
| 11696071 |
| 11696072 |
| 11696073 |
| 11696076 |
| 11696077 |
| 11711488 |
| 11716165 |
| 11716166 |
| 11716173 |
| 11717243 |
| 11723210 |
| 11748054 |
| 11748349 |
| 11773333 |
| 11788360 |
| 11790520 |
| 11806880 |
| 11807189 |
| 11818762 |
| 11829107 |
| 11832528 |
| 11836975 |
| 11839458 |
| 11843310 |
| 11869425 |
| 11869426 |
| 11869427 |
| 11869430 |
| 11896038 |
| 11903855 |
| 11903857 |
| 11903859 |
| 11903860 |
| 11903861 |
| 11903862 |
| 11903863 |
| 11927702 |
| 11936529 |
| 11936530 |
| 11936531 |
| 11959801 |
| 11961424 |
| 11964587 |
| 11964595 |
| 11967716 |
| 11971059 |
| 11978918 |
| 11978919 |
| 11978920 |
| 11991909 |
| 11998993 |
| 12015370 |
| 12015374 |
| 12015376 |
| 12023429 |
| 12028474 |
| 12028475 |
| 12028476 |
| 12028477 |
| 12028480 |
| 12028482 |
| 12034649 |
| 12037222 |
| 12040077 |
| 12070197 |
| 12096203 |
| 12097521 |
| 12097766 |
| 12097767 |
| 12105352 |
| 12107292 |
| 12121866 |
| 12121867 |
| 12138072 |
| 12149535 |
| 12151563 |
| 12166562 |
| 12166563 |
| 12166564 |
| 12183486 |
| 12183506 |
| 12193515 |
| 12193650 |
| 12198007 |
| 12205139 |
| 12205231 |
| 12212980 |
| 12212981 |
| 12220314 |
| 12220315 |
| 12220319 |
| 12220321 |
| 12220322 |
| 12220323 |
| 12225703 |
| 12234953 |
| 12235037 |
| 12236607 |
| 12242196 |
| 12357140 |
| 12358352 |
| 12358354 |
| 12388174 |
| 12390928 |
| 12391093 |
| 12391097 |
| 12391099 |
| 12391120 |
| 12391386 |
| 12399283 |
| 12405615 |
| 12431858 |
| 12433858 |
| 12433860 |
| 12438461 |
| 12438490 |
| 12449179 |
| 12449180 |
| 12449181 |
| 12454300 |
| 12462350 |
| 12464094 |
| 12464096 |
| 12464097 |
| 12464098 |
| 12464101 |
| 12464102 |
| 12464103 |
| 12464458 |
| 12464461 |
| 12480970 |
| 12486189 |
| 12496135 |
| 12503711 |
| 12503712 |
| 12506338 |
| 12509495 |
| 12514164 |
| 12515745 |
| 12529281 |
| 12538317 |
| 12553592 |
| 12556069 |
| 12576405 |
| 12576408 |
| 12578876 |
| 12589388 |
| 12589390 |
| 12598632 |
| 12603781 |
| 12603782 |
| 12603783 |
| 12603785 |
| 12603787 |
| 12603788 |
| 12603789 |
| 12608436 |
| 12608437 |
| 12608438 |
| 12615558 |
| 12615559 |
| 12615790 |
| 12627872 |
| 12629537 |
| 12629540 |
| 12642360 |
| 12656666 |
| 12659290 |
| 12662010 |
| 12662011 |
| 12662012 |
| 12695297 |
| 12700309 |
| 12713019 |
| 12716752 |
| 12743002 |
| 12753345 |
| 12753347 |
| 12753348 |
| 12753351 |
| 12753353 |
| 12762357 |
| 12766642 |
| 12793998 |
| 12795811 |
| 12797491 |
| 12799616 |
| 12807995 |
| 12810790 |
| 12812993 |
| 12819215 |
| 12857768 |
| 12865895 |
| 12882466 |
| 12888546 |
| 12897029 |
| 12904452 |
| 12907416 |
| 12932084 |
| 12937025 |
| 12938843 |
| 12938844 |
| 12941056 |
| 12941057 |
| 12941058 |
| 12941060 |
| 12941061 |
| 12941063 |
| 12947143 |
| 12952256 |
| 12952257 |
| 12952844 |
| 12955096 |
| 12959951 |
| 12963804 |
| 12967921 |
| 14555669 |
| 14567523 |
| 14573724 |
| 14577838 |
| 14578367 |
| 14582920 |
| 14585110 |
| 14604221 |
| 14621092 |
| 14625220 |
| 14629777 |
| 14630625 |
| 14633238 |
| 14633242 |
| 14633244 |
| 14633245 |
| 14633280 |
| 14634196 |
| 14639141 |
| 14645485 |
| 14647485 |
| 14654457 |
| 14657548 |
| 14670764 |
| 14670769 |
| 14671044 |
| 14672898 |
| 14680082 |
| 14680083 |
| 14694248 |
| 14694250 |
| 14732791 |
| 14736626 |
| 14738229 |
| 14738230 |
| 14738231 |
| 14766981 |
| 14871884 |
| 14872107 |
| 14960432 |
| 14979499 |
| 14979500 |
| 14979501 |
| 14981054 |
| 14990552 |
| 14996032 |
| 14996034 |
| 14996037 |
| 14996038 |
| 14996039 |
| 14996040 |
| 14996041 |
| 15007496 |
| 15031568 |
| 15039509 |
| 15040803 |
| 15044698 |
| 15047958 |
| 15054059 |
| 15054065 |
| 15065685 |
| 15065833 |
| 15068660 |
| 15070419 |
| 15080780 |
| 15083761 |
| 15087614 |
| 15096280 |
| 15115546 |
| 15120882 |
| 15120883 |
| 15133345 |
| 15139072 |
| 15140927 |
| 15142836 |
| 15163350 |
| 15170032 |
| 15175089 |
| 15175091 |
| 15175093 |
| 15175095 |
| 15175096 |
| 15175097 |
| 15176689 |
| 15187983 |
| 15191779 |
| 15194678 |
| 15212122 |
| 15219003 |
| 15234956 |
| 15249509 |
| 15257305 |
| 15257995 |
| 15269066 |
| 15288160 |
| 15293607 |
| 15293639 |
| 15295020 |
| 15302984 |
| 15308479 |
| 15316212 |
| 15316213 |
| 15317668 |
| 15332396 |
| 15332397 |
| 15332398 |
| 15333855 |
| 15339256 |
| 15339258 |
| 15339259 |
| 15339260 |
| 15339261 |
| 15339262 |
| 15339263 |
| 15353412 |
| 15356341 |
| 15358704 |
| 15362959 |
| 15369129 |
| 15448124 |
| 15453551 |
| 15454655 |
| 15459085 |
| 15467327 |
| 15467328 |
| 15471971 |
| 15479904 |
| 15492313 |
| 15509816 |
| 15516674 |
| 15521844 |
| 15525784 |
| 15538933 |
| 15548552 |
| 15560763 |
| 15560764 |
| 15560765 |
| 15560766 |
| 15560769 |
| 15560771 |
| 15560772 |
| 15560773 |
| 15568121 |
| 15569118 |
| 15572542 |
| 15572543 |
| 15576888 |
| 15576889 |
| 15577276 |
| 15579578 |
| 15583127 |
| 15588154 |
| 15602499 |
| 15602591 |
| 15611605 |
| 15618315 |
| 15618323 |
| 15627867 |
| 15627868 |
| 15634777 |
| 15639757 |
| 15640325 |
| 15640326 |
| 15642501 |
| 15642774 |
| 15644131 |
| 15677527 |
| 15677579 |
| 15677733 |
| 15679759 |
| 15682476 |
| 15683194 |
| 15684300 |
| 15684301 |
| 15687230 |
| 15687326 |
| 15699282 |
| 15703253 |
| 15705609 |
| 15714224 |
| 15714228 |
| 15718408 |
| 15733510 |
| 15738297 |
| 15738298 |
| 15743327 |
| 15743328 |
| 15743336 |
| 15743337 |
| 15743538 |
| 15749721 |
| 15750038 |
| 15779501 |
| 15790683 |
| 15794028 |
| 15802342 |
| 15817803 |
| 15818653 |
| 15823448 |
| 15824523 |
| 15824524 |
| 15837952 |
| 15840682 |
| 15854175 |
| 15860719 |
| 15860720 |
| 15863638 |
| 15866856 |
| 15866860 |
| 15878488 |
| 15879419 |
| 15888642 |
| 15890999 |
| 15910508 |
| 15910509 |
| 15910511 |
| 15910512 |
| 15910514 |
| 15910515 |
| 15910516 |
| 15914575 |
| 15923252 |
| 15939247 |
| 15942297 |
| 15948818 |
| 15954201 |
| 15958722 |
| 15961535 |
| 15963401 |
| 15983234 |
| 15994243 |
| 15994252 |
| 16002438 |
| 16009798 |
| 16010529 |
| 16024243 |
| 16024516 |
| 16049036 |
| 16055877 |
| 16061712 |
| 16079371 |
| 16085213 |
| 16088283 |
| 16097999 |
| 16105851 |
| 16109727 |
| 16112658 |
| 16118049 |
| 16120097 |
| 16120098 |
| 16120099 |
| 16120100 |
| 16120101 |
| 16120103 |
| 16120108 |
| 16124064 |
| 16126716 |
| 16129972 |
| 16130027 |
| 16135475 |
| 16140228 |
| 16140233 |
| 16141374 |
| 16141444 |
| 16144846 |
| 16146572 |
| 16148240 |
| 16171278 |
| 16179400 |
| 16192452 |
| 16192980 |
| 16197495 |
| 16204600 |
| 16204601 |
| 16204772 |
| 16204778 |
| 16204780 |
| 16204784 |
| 16204797 |
| 16205042 |
| 16210668 |
| 16210883 |
| 16219453 |
| 16221767 |
| 16230426 |
| 16237393 |
| 16246540 |
| 16251217 |
| 16251267 |
| 16254055 |
| 16267074 |
| 16272771 |
| 16278081 |
| 16278367 |
| 16282003 |
| 16282178 |
| 16282549 |
| 16286412 |
| 16286569 |
| 16287708 |
| 16289589 |
| 16295210 |
| 16299414 |
| 16301549 |
| 16306160 |
| 16306164 |
| 16318915 |
| 16337115 |
| 16339020 |
| 16339311 |
| 16339767 |
| 16339798 |
| 16342098 |
| 16352624 |
| 16357087 |
| 16361457 |
| 16361593 |
| 16362680 |
| 16362683 |
| 16364133 |
| 16364134 |
| 16364136 |
| 16364137 |
| 16364138 |
| 16364139 |
| 16364141 |
| 16364142 |
| 16364144 |
| 16364145 |
| 16364146 |
| 16377643 |
| 16380239 |
| 16380551 |
| 16384630 |
| 16387944 |
| 16387945 |
| 16388943 |
| 16396849 |
| 16399781 |
| 16401347 |
| 16412864 |
| 16417639 |
| 16424443 |
| 16429591 |
| 16439671 |
| 16439714 |
| 16452329 |
| 16452587 |
| 16452588 |
| 16460564 |
| 16467311 |
| 16481379 |
| 16489705 |
| 16489997 |
| 16489999 |
| 16490000 |
| 16490001 |
| 16490003 |
| 16490004 |
| 16490006 |
| 16490007 |
| 16490008 |
| 16490766 |
| 16495472 |
| 16497687 |
| 16507857 |
| 16507858 |
| 16510459 |
| 16510622 |
| 16510628 |
| 16510665 |
| 16524482 |
| 16532711 |
| 16537671 |
| 16541085 |
| 16543515 |
| 16543543 |
| 16545553 |
| 16546407 |
| 16549947 |
| 16556620 |
| 16571610 |
| 16580533 |
| 16585081 |
| 16585083 |
| 16585288 |
| 16585410 |
| 16597649 |
| 16627860 |
| 16636288 |
| 16641120 |
| 16645689 |
| 16648209 |
| 16675631 |
| 16676780 |
| 16690976 |
| 16704564 |
| 16704566 |
| 16704567 |
| 16704569 |
| 16704570 |
| 16704571 |
| 16704572 |
| 16704573 |
| 16704574 |
| 16704575 |
| 16704577 |
| 16704578 |
| 16707395 |
| 16707396 |
| 16707482 |
| 16709652 |
| 16709938 |
| 16740591 |
| 16772358 |
| 16818551 |
| 16818554 |
| 16822972 |
| 16825526 |
| 16829553 |
| 16840653 |
| 16845599 |
| 16849364 |
| 16861714 |
| 16870662 |
| 16872821 |
| 16880368 |
| 16880772 |
| 16882769 |
| 16885414 |
| 16888274 |
| 16894010 |
| 16895254 |
| 16908126 |
| 16911025 |
| 16911026 |
| 16911027 |
| 16911028 |
| 16911029 |
| 16911030 |
| 16911031 |
| 16911033 |
| 16911034 |
| 16911035 |
| 16911037 |
| 16912060 |
| 16912152 |
| 16914506 |
| 16919928 |
| 16924359 |
| 16928705 |
| 16928708 |
| 16928713 |
| 16933960 |
| 16936703 |
| 16937757 |
| 16940468 |
| 16943553 |
| 16950965 |
| 16951007 |
| 16952333 |
| 16956914 |
| 16979688 |
| 16990963 |
| 16996309 |
| 17003142 |
| 17005578 |
| 17005584 |
| 17008033 |
| 17008440 |
| 17011823 |
| 17015501 |
| 17035531 |
| 17041010 |
| 17049441 |
| 17050561 |
| 17052349 |
| 17053102 |
| 17071847 |
| 17072037 |
| 17072038 |
| 17072068 |
| 17072857 |
| 17078884 |
| 17079578 |
| 17082238 |
| 17109739 |
| 17110530 |
| 17117964 |
| 17118091 |
| 17118092 |
| 17118093 |
| 17118094 |
| 17118097 |
| 17118099 |
| 17118100 |
| 17118103 |
| 17121868 |
| 17122092 |
| 17136879 |
| 17137777 |
| 17158146 |
| 17166646 |
| 17169161 |
| 17172634 |
| 17178502 |
| 17185213 |
| 17185498 |
| 17198546 |
| 17200244 |
| 17200274 |
| 17203011 |
| 17204492 |
| 17204730 |
| 17223328 |
| 17235125 |
| 17235126 |
| 17235580 |
| 17244938 |
| 17250898 |
| 17251229 |
| 17251313 |
| 17251319 |
| 17255543 |
| 17258090 |
| 17261645 |
| 17268123 |
| 17272582 |
| 17272622 |
| 17276180 |
| 17277346 |
| 17284739 |
| 17301093 |
| 17309758 |
| 17309760 |
| 17309761 |
| 17309762 |
| 17309765 |
| 17309766 |
| 17309768 |
| 17309770 |
| 17309772 |
| 17322492 |
| 17329439 |
| 17342210 |
| 17353437 |
| 17353512 |
| 17360729 |
| 17360730 |
| 17363123 |
| 17363769 |
| 17371108 |
| 17371117 |
| 17389258 |
| 17395627 |
| 17397701 |
| 17398152 |
| 17400590 |
| 17400602 |
| 17400731 |
| 17400929 |
| 17406665 |
| 17410279 |
| 17412778 |
| 17412783 |
| 17412784 |
| 17413053 |
| 17415135 |
| 17433953 |
| 17440612 |
| 17442693 |
| 17448652 |
| 17449637 |
| 17459896 |
| 17464380 |
| 17470670 |
| 17473079 |
| 17473096 |
| 17478489 |
| 17483481 |
| 17488863 |
| 17505053 |
| 17512359 |
| 17517619 |
| 17522024 |
| 17531413 |
| 17541017 |
| 17542943 |
| 17542946 |
| 17542947 |
| 17542948 |
| 17542949 |
| 17542951 |
| 17542952 |
| 17542954 |
| 17543813 |
| 17545369 |
| 17547910 |
| 17552381 |
| 17554084 |
| 17555420 |
| 17556419 |
| 17557438 |
| 17557769 |
| 17561593 |
| 17561598 |
| 17562959 |
| 17567673 |
| 17569763 |
| 17570059 |
| 17573370 |
| 17573448 |
| 17573498 |
| 17580591 |
| 17580594 |
| 17580595 |
| 17580597 |
| 17580599 |
| 17580600 |
| 17580601 |
| 17580602 |
| 17580603 |
| 17584779 |
| 17585061 |
| 17597542 |
| 17600160 |
| 17615093 |
| 17615417 |
| 17619774 |
| 17626107 |
| 17626108 |
| 17627965 |
| 17634447 |
| 17635854 |
| 17641220 |
| 17667845 |
| 17670944 |
| 17673558 |
| 17676331 |
| 17682651 |
| 17682652 |
| 17682653 |
| 17682654 |
| 17682661 |
| 17682662 |
| 17682664 |
| 17689142 |
| 17690117 |
| 17690200 |
| 17694722 |
| 17694723 |
| 17694727 |
| 17694728 |
| 17698504 |
| 17699290 |
| 17699426 |
| 17702264 |
| 17702267 |
| 17702268 |
| 17702269 |
| 17702270 |
| 17702271 |
| 17702272 |
| 17702273 |
| 17702733 |
| 17716271 |
| 17716272 |
| 17716273 |
| 17716275 |
| 17716276 |
| 17716277 |
| 17716278 |
| 17716279 |
| 17716280 |
| 17716281 |
| 17716283 |
| 17724256 |
| 17765353 |
| 17766521 |
| 17766685 |
| 17767677 |
| 17785629 |
| 17803007 |
| 17803008 |
| 17803009 |
| 17803010 |
| 17803011 |
| 17803014 |
| 17823298 |
| 17823448 |
| 17825610 |
| 17825619 |
| 17848998 |
| 17855122 |
| 17855728 |
| 17868655 |
| 17869080 |
| 17890740 |
| 17890744 |
| 17890750 |
| 17892929 |
| 17898013 |
| 17898015 |
| 17903308 |
| 17903628 |
| 17908734 |
| 17908765 |
| 17910380 |
| 17910381 |
| 17910382 |
| 17910384 |
| 17910386 |
| 17910387 |
| 17910389 |
| 17910390 |
| 17910393 |
| 17910395 |
| 17911092 |
| 17916615 |
| 17923535 |
| 17926333 |
| 17928550 |
| 17945544 |
| 17947257 |
| 17951025 |
| 17956928 |
| 17957147 |
| 17958471 |
| 17964738 |
| 17967815 |
| 17967978 |
| 17969458 |
| 17969462 |
| 17969464 |
| 17969465 |
| 17969466 |
| 17969468 |
| 17969470 |
| 17969471 |
| 17969472 |
| 17971337 |
| 17974734 |
| 17974739 |
| 17974740 |
| 17984430 |
| 17992551 |
| 17993037 |
| 17993039 |
| 17993040 |
| 17993043 |
| 17993045 |
| 17993046 |
| 18000060 |
| 18003825 |
| 18006903 |
| 18024981 |
| 18032443 |
| 18036078 |
| 18036079 |
| 18036080 |
| 18036082 |
| 18036084 |
| 18036085 |
| 18036086 |
| 18036087 |
| 18036088 |
| 18036089 |
| 18037670 |
| 18041478 |
| 18041482 |
| 18041483 |
| 18041484 |
| 18041485 |
| 18041486 |
| 18041487 |
| 18041490 |
| 18041491 |
| 18043740 |
| 18046019 |
| 18048862 |
| 18049292 |
| 18053240 |
| 18055094 |
| 18056138 |
| 18056139 |
| 18056144 |
| 18058583 |
| 18070833 |
| 18071053 |
| 18073231 |
| 18073234 |
| 18075410 |
| 18077474 |
| 18078723 |
| 18079168 |
| 18162715 |
| 18164280 |
| 18172212 |
| 18174542 |
| 18176336 |
| 18184678 |
| 18184681 |
| 18192289 |
| 18192301 |
| 18198351 |
| 18198363 |
| 18198800 |
| 18198801 |
| 18198802 |
| 18198804 |
| 18198805 |
| 18198806 |
| 18203694 |
| 18204112 |
| 18204121 |
| 18204141 |
| 18204146 |
| 18204181 |
| 18209735 |
| 18215538 |
| 18216135 |
| 18220078 |
| 18220080 |
| 18220082 |
| 18220083 |
| 18220085 |
| 18220087 |
| 18226948 |
| 18229986 |
| 18246972 |
| 18246974 |
| 18246975 |
| 18246976 |
| 18246978 |
| 18246979 |
| 18246982 |
| 18246984 |
| 18246985 |
| 18246986 |
| 18246987 |
| 18246988 |
| 18246989 |
| 18248944 |
| 18250091 |
| 18256066 |
| 18256067 |
| 18258619 |
| 18263678 |
| 18268935 |
| 18269740 |
| 18272662 |
| 18274265 |
| 18274267 |
| 18274270 |
| 18274273 |
| 18274275 |
| 18274276 |
| 18275549 |
| 18275550 |
| 18275552 |
| 18275553 |
| 18275554 |
| 18275556 |
| 18275557 |
| 18275559 |
| 18276938 |
| 18276939 |
| 18281697 |
| 18287107 |
| 18292297 |
| 18293151 |
| 18307774 |
| 18310650 |
| 18321246 |
| 18321934 |
| 18321935 |
| 18326715 |
| 18328671 |
| 18337406 |
| 18343647 |
| 18343856 |
| 18343862 |
| 18347208 |
| 18350958 |
| 18350959 |
| 18350960 |
| 18350963 |
| 18350964 |
| 18362052 |
| 18362291 |
| 18362594 |
| 18363307 |
| 18363314 |
| 18363315 |
| 18363316 |
| 18363317 |
| 18363319 |
| 18363320 |
| 18367762 |
| 18384588 |
| 18389288 |
| 18391138 |
| 18396419 |
| 18398808 |
| 18401642 |
| 18403451 |
| 18410166 |
| 18413499 |
| 18413992 |
| 18414674 |
| 18415718 |
| 18417644 |
| 18420722 |
| 18422508 |
| 18423009 |
| 18431116 |
| 18431117 |
| 18434020 |
| 18437259 |
| 18448486 |
| 18450864 |
| 18457239 |
| 18457240 |
| 18457241 |
| 18457242 |
| 18457243 |
| 18457244 |
| 18457245 |
| 18458174 |
| 18459154 |
| 18468307 |
| 18468308 |
| 18468309 |
| 18468310 |
| 18468311 |
| 18469506 |
| 18474105 |
| 18475156 |
| 18480104 |
| 18480189 |
| 18482106 |
| 18482107 |
| 18482108 |
| 18482109 |
| 18482110 |
| 18482111 |
| 18482297 |
| 18482867 |
| 18483003 |
| 18483473 |
| 18484964 |
| 18487566 |
| 18495885 |
| 18495886 |
| 18497601 |
| 18499780 |
| 18503512 |
| 18503513 |
| 18504636 |
| 18505619 |
| 18508832 |
| 18511023 |
| 18513353 |
| 18515807 |
| 18516635 |
| 18517030 |
| 18517031 |
| 18517034 |
| 18517035 |
| 18517036 |
| 18517041 |
| 18517042 |
| 18517043 |
| 18517044 |
| 18519486 |
| 18522716 |
| 18523125 |
| 18534222 |
| 18534224 |
| 18544879 |
| 18544883 |
| 18547374 |
| 18548823 |
| 18548825 |
| 18548826 |
| 18548827 |
| 18548831 |
| 18548836 |
| 18550611 |
| 18551328 |
| 18561896 |
| 18564297 |
| 18564298 |
| 18566693 |
| 18576297 |
| 18577286 |
| 18579365 |
| 18579368 |
| 18579542 |
| 18582607 |
| 18582898 |
| 18585071 |
| 18585104 |
| 18595433 |
| 18595434 |
| 18595436 |
| 18595437 |
| 18595959 |
| 18596620 |
| 18598851 |
| 18606530 |
| 18606532 |
| 18614178 |
| 18616688 |
| 18617308 |
| 18617382 |
| 18617656 |
| 18624714 |
| 18626095 |
| 18628443 |
| 18632776 |
| 18635832 |
| 18640017 |
| 18641111 |
| 18644799 |
| 18647950 |
| 18652089 |
| 18652091 |
| 18652092 |
| 18652093 |
| 18652094 |
| 18652095 |
| 18652096 |
| 18652097 |
| 18652941 |
| 18657881 |
| 18658114 |
| 18662202 |
| 18663241 |
| 18663242 |
| 18664587 |
| 18664997 |
| 18667284 |
| 18667623 |
| 18673569 |
| 18676503 |
| 18676540 |
| 18677716 |
| 18678797 |
| 18684853 |
| 18687412 |
| 18691289 |
| 18691437 |
| 18693226 |
| 18697817 |
| 18698363 |
| 18703381 |
| 18707765 |
| 18710420 |
| 18711015 |
| 18711110 |
| 18714207 |
| 18714778 |
| 18714779 |
| 18714780 |
| 18714781 |
| 18714782 |
| 18714785 |
| 18714786 |
| 18714787 |
| 18714788 |
| 18714789 |
| 18714790 |
| 18716203 |
| 18752997 |
| 18753730 |
| 18754839 |
| 18757699 |
| 18759758 |
| 18761599 |
| 18762495 |
| 18762497 |
| 18763420 |
| 18763421 |
| 18763423 |
| 18763424 |
| 18763426 |
| 18763427 |
| 18763428 |
| 18763429 |
| 18765482 |
| 18765561 |
| 18778837 |
| 18779282 |
| 18782953 |
| 18786977 |
| 18787652 |
| 18787720 |
| 18788647 |
| 18788648 |
| 18788650 |
| 18788651 |
| 18788653 |
| 18788655 |
| 18795985 |
| 18799950 |
| 18801037 |
| 18804245 |
| 18809152 |
| 18810719 |
| 18811993 |
| 18813042 |
| 18815373 |
| 18817803 |
| 18819173 |
| 18823289 |
| 18823428 |
| 18828142 |
| 18828917 |
| 18829212 |
| 18829972 |
| 18832089 |
| 18832332 |
| 18835917 |
| 18838624 |
| 18842606 |
| 18844819 |
| 18845553 |
| 18850259 |
| 18853307 |
| 18853696 |
| 18853698 |
| 18853699 |
| 18853702 |
| 18853703 |
| 18853709 |
| 18853931 |
| 18853933 |
| 18853937 |
| 18853938 |
| 18853939 |
| 18853940 |
| 18853941 |
| 18853943 |
| 18854708 |
| 18855824 |
| 18925322 |
| 18927265 |
| 18930805 |
| 18936766 |
| 18945686 |
| 18946511 |
| 18948445 |
| 18948968 |
| 18949106 |
| 18952563 |
| 18971289 |
| 18971479 |
| 18972392 |
| 18977973 |
| 18990675 |
| 18996049 |
| 19001199 |
| 19007876 |
| 19008237 |
| 19008239 |
| 19010989 |
| 19011148 |
| 19012722 |
| 19012723 |
| 19014070 |
| 19014071 |
| 19014072 |
| 19014073 |
| 19014074 |
| 19014075 |
| 19014076 |
| 19014077 |
| 19015202 |
| 19015401 |
| 19021849 |
| 19021850 |
| 19021851 |
| 19021852 |
| 19021856 |
| 19022640 |
| 19026314 |
| 19028674 |
| 19040608 |
| 19041689 |
| 19047218 |
| 19047224 |
| 19047310 |
| 19047314 |
| 19056602 |
| 19058790 |
| 19059729 |
| 19066308 |
| 19073294 |
| 19076157 |
| 19087899 |
| 19088412 |
| 19090322 |
| 19090324 |
| 19090326 |
| 19090327 |
| 19090328 |
| 19090331 |
| 19090952 |
| 19090953 |
| 19091084 |
| 19091126 |
| 19092240 |
| 19098064 |
| 19104169 |
| 19108665 |
| 19109537 |
| 19110880 |
| 19110882 |
| 19110883 |
| 19110884 |
| 19110886 |
| 19110887 |
| 19110888 |
| 19116797 |
| 19117855 |
| 19118118 |
| 19118266 |
| 19118273 |
| 19118766 |
| 19126856 |
| 19128903 |
| 19129293 |
| 19132641 |
| 19133287 |
| 19136281 |
| 19137236 |
| 19138880 |
| 19139047 |
| 19144652 |
| 19144755 |
| 19147446 |
| 19148690 |
| 19155746 |
| 19155748 |
| 19164756 |
| 19167642 |
| 19168380 |
| 19171641 |
| 19185287 |
| 19189775 |
| 19189777 |
| 19189778 |
| 19189781 |
| 19189782 |
| 19189783 |
| 19189862 |
| 19194375 |
| 19196901 |
| 19197217 |
| 19203874 |
| 19204069 |
| 19208245 |
| 19208264 |
| 19213784 |
| 19213789 |
| 19213794 |
| 19216936 |
| 19217270 |
| 19218636 |
| 19218642 |
| 19218687 |
| 19218688 |
| 19218693 |
| 19218703 |
| 19218761 |
| 19220560 |
| 19222324 |
| 19223282 |
| 19225059 |
| 19228985 |
| 19228987 |
| 19232575 |
| 19238800 |
| 19238805 |
| 19238806 |
| 19238807 |
| 19238808 |
| 19238809 |
| 19238811 |
| 19238812 |
| 19238813 |
| 19238814 |
| 19238815 |
| 19240794 |
| 19246183 |
| 19250172 |
| 19250173 |
| 19250174 |
| 19250175 |
| 19250176 |
| 19251583 |
| 19251866 |
| 19255295 |
| 19255447 |
| 19261296 |
| 19262465 |
| 19264976 |
| 19266584 |
| 19277618 |
| 19282237 |
| 19286627 |
| 19293692 |
| 19293693 |
| 19293694 |
| 19294949 |
| 19294950 |
| 19294951 |
| 19294952 |
| 19294953 |
| 19294954 |
| 19294956 |
| 19294957 |
| 19294958 |
| 19294959 |
| 19294960 |
| 19294963 |
| 19297363 |
| 19299406 |
| 19301532 |
| 19302341 |
| 19302342 |
| 19302344 |
| 19304118 |
| 19317376 |
| 19319586 |
| 19322104 |
| 19324954 |
| 19326184 |
| 19330249 |
| 19330583 |
| 19336360 |
| 19336376 |
| 19336377 |
| 19336590 |
| 19337560 |
| 19348959 |
| 19349237 |
| 19357147 |
| 19357151 |
| 19360494 |
| 19361854 |
| 19365641 |
| 19375670 |
| 19379769 |
| 19379771 |
| 19384418 |
| 19386910 |
| 19393404 |
| 19396527 |
| 19399951 |
| 19403012 |
| 19403479 |
| 19403493 |
| 19404716 |
| 19407048 |
| 19413142 |
| 19413143 |
| 19413146 |
| 19413147 |
| 19413148 |
| 19413152 |
| 19424440 |
| 19426521 |
| 19427354 |
| 19427574 |
| 19444752 |
| 19450933 |
| 19459802 |
| 19460089 |
| 19465319 |
| 19465743 |
| 19466746 |
| 19479080 |
| 19480226 |
| 19480228 |
| 19480229 |
| 19480230 |
| 19480231 |
| 19480232 |
| 19480234 |
| 19480236 |
| 19482732 |
| 19486363 |
| 19491110 |
| 19491151 |
| 19493297 |
| 19493298 |
| 19494088 |
| 19496951 |
| 19502617 |
| 19505495 |
| 19505769 |
| 19506175 |
| 19516903 |
| 19520155 |
| 19521652 |
| 19521723 |
| 19533192 |
| 19534799 |
| 19536428 |
| 19542208 |
| 19542260 |
| 19544748 |
| 19544750 |
| 19544751 |
| 19544754 |
| 19544755 |
| 19544756 |
| 19544758 |
| 19546306 |
| 19546416 |
| 19548627 |
| 19549077 |
| 19549078 |
| 19549678 |
| 19549821 |
| 19552702 |
| 19552703 |
| 19553435 |
| 19560866 |
| 19564269 |
| 19564298 |
| 19564303 |
| 19566894 |
| 19567491 |
| 19567497 |
| 19573081 |
| 19574323 |
| 19574325 |
| 19574334 |
| 19574335 |
| 19574336 |
| 19578392 |
| 19581829 |
| 19585672 |
| 19590434 |
| 19604370 |
| 19606092 |
| 19608587 |
| 19608589 |
| 19615098 |
| 19616140 |
| 19616141 |
| 19616171 |
| 19617235 |
| 19625733 |
| 19626593 |
| 19627224 |
| 19631989 |
| 19635063 |
| 19639747 |
| 19639748 |
| 19639752 |
| 19639754 |
| 19639756 |
| 19639757 |
| 19639758 |
| 19641160 |
| 19643769 |
| 19643928 |
| 19643936 |
| 19644043 |
| 19645961 |
| 19645963 |
| 19645964 |
| 19645965 |
| 19645966 |
| 19645969 |
| 19648298 |
| 19648462 |
| 19651569 |
| 19651664 |
| 19651919 |
| 19652849 |
| 19656277 |
| 19663929 |
| 19663998 |
| 19665729 |
| 19665944 |
| 19666692 |
| 19672009 |
| 19672013 |
| 19672177 |
| 19675059 |
| 19680805 |
| 19682231 |
| 19682242 |
| 19683953 |
| 19684146 |
| 19684340 |
| 19685943 |
| 19686237 |
| 19688045 |
| 19691473 |
| 19699148 |
| 19702431 |
| 19702469 |
| 19702788 |
| 19706591 |
| 19707599 |
| 19720267 |
| 19720777 |
| 19721845 |
| 19725249 |
| 19725251 |
| 19725253 |
| 19725256 |
| 19725257 |
| 19725259 |
| 19725260 |
| 19725261 |
| 19725262 |
| 19732315 |
| 19732316 |
| 19732318 |
| 19735444 |
| 19743760 |
| 19745203 |
| 19748312 |
| 19750916 |
| 19750919 |
| 19750920 |
| 19750921 |
| 19750922 |
| 19750926 |
| 19750928 |
| 19751424 |
| 19752724 |
| 19761534 |
| 19762565 |
| 19765205 |
| 19765206 |
| 19766936 |
| 19773369 |
| 19774216 |
| 19775835 |
| 19776261 |
| 19779153 |
| 19780473 |
| 19786163 |
| 19786682 |
| 19787489 |
| 19793160 |
| 19793356 |
| 19797130 |
| 19797158 |
| 19799778 |
| 19805747 |
| 19806214 |
| 19808720 |
| 19814603 |
| 19820075 |
| 19840241 |
| 19840242 |
| 19840243 |
| 19840960 |
| 19841107 |
| 19843648 |
| 19845847 |
| 19845849 |
| 19846546 |
| 19848355 |
| 19848356 |
| 19848357 |
| 19848358 |
| 19848361 |
| 19848362 |
| 19848363 |
| 19848364 |
| 19848365 |
| 19848367 |
| 19850211 |
| 19854036 |
| 19855839 |
| 19857033 |
| 19858078 |
| 19858153 |
| 19874580 |
| 19874994 |
| 19875421 |
| 19877745 |
| 19878448 |
| 19878450 |
| 19879459 |
| 19879556 |
| 19884499 |
| 19884558 |
| 19885853 |
| 19888304 |
| 19890014 |
| 19890169 |
| 19895422 |
| 19895423 |
| 19895425 |
| 19907004 |
| 19910178 |
| 19910332 |
| 19912509 |
| 19912511 |
| 19912512 |
| 19917590 |
| 19922343 |
| 19923203 |
| 19928383 |
| 19928385 |
| 19928391 |
| 19933407 |
| 19933917 |
| 19936804 |
| 19937049 |
| 19940101 |
| 19943990 |
| 19955499 |
| 19955705 |
| 19958890 |
| 19959590 |
| 19960521 |
| 19960637 |
| 19960639 |
| 19960641 |
| 19960643 |
| 19960644 |
| 19961023 |
| 19961024 |
| 19961025 |
| 19961026 |
| 19961027 |
| 19961029 |
| 19961030 |
| 19961031 |
| 19961032 |
| 19961033 |
| 19962939 |
| 19965541 |
| 19968005 |
| 19968007 |
| 19968008 |
| 19968009 |
| 19968010 |
| 19968011 |
| 19968013 |
| 19968014 |
| 19968040 |
| 19968042 |
| 19968043 |
| 19968044 |
| 19968047 |
| 19968048 |
| 19968881 |
| 19995884 |
| 19997572 |
| 20004301 |
| 20004888 |
| 20005774 |
| 20007746 |
| 20009370 |
| 20010134 |
| 20016520 |
| 20019118 |
| 20019340 |
| 20019675 |
| 20026494 |
| 20028364 |
| 20032012 |
| 20033109 |
| 20034719 |
| 20037709 |
| 20040036 |
| 20040038 |
| 20041591 |
| 20041592 |
| 20041593 |
| 20041594 |
| 20041596 |
| 20041598 |
| 20042689 |
| 20043249 |
| 20049334 |
| 20050992 |
| 20050993 |
| 20050995 |
| 20050996 |
| 20050997 |
| 20051046 |
| 20057899 |
| 20058567 |
| 20075004 |
| 20075057 |
| 20075361 |
| 20075435 |
| 20077997 |
| 20078008 |
| 20078829 |
| 20079978 |
| 20080444 |
| 20082240 |
| 20083371 |
| 20083430 |
| 20083521 |
| 20083800 |
| 20084368 |
| 20090370 |
| 20093640 |
| 20093641 |
| 20099452 |
| 20105456 |
| 20110399 |
| 20120620 |
| 20120621 |
| 20120622 |
| 20120625 |
| 20120627 |
| 20120628 |
| 20120629 |
| 20122554 |
| 20123412 |
| 20123846 |
| 20123859 |
| 20129934 |
| 20133380 |
| 20134039 |
| 20138492 |
| 20138579 |
| 20139658 |
| 20140099 |
| 20142493 |
| 20149067 |
| 20149068 |
| 20149069 |
| 20156343 |
| 20156725 |
| 20156767 |
| 20157410 |
| 20159778 |
| 20159829 |
| 20160141 |
| 20164352 |
| 20175398 |
| 20175399 |
| 20175401 |
| 20175402 |
| 20175404 |
| 20175405 |
| 20175406 |
| 20175407 |
| 20175408 |
| 20175409 |
| 20175411 |
| 20179368 |
| 20183719 |
| 20183721 |
| 20183723 |
| 20185739 |
| 20186573 |
| 20187847 |
| 20190331 |
| 20191392 |
| 20191932 |
| 20191934 |
| 20191935 |
| 20191936 |
| 20191937 |
| 20191938 |
| 20191939 |
| 20191940 |
| 20191941 |
| 20195475 |
| 20199233 |
| 20199687 |
| 20200778 |
| 20202887 |
| 20203242 |
| 20205564 |
| 20205705 |
| 20206074 |
| 20209158 |
| 20217365 |
| 20221849 |
| 20222990 |
| 20223916 |
| 20223918 |
| 20223993 |
| 20224943 |
| 20226732 |
| 20230084 |
| 20231014 |
| 20299536 |
| 20299626 |
| 20303248 |
| 20304429 |
| 20304755 |
| 20307395 |
| 20333306 |
| 20335353 |
| 20337186 |
| 20337189 |
| 20337190 |
| 20337191 |
| 20337193 |
| 20337196 |
| 20337197 |
| 20337198 |
| 20337903 |
| 20337904 |
| 20337905 |
| 20337906 |
| 20339144 |
| 20339305 |
| 20347414 |
| 20348220 |
| 20350515 |
| 20351237 |
| 20352547 |
| 20354007 |
| 20354236 |
| 20357041 |
| 20357381 |
| 20362503 |
| 20363668 |
| 20370475 |
| 20371466 |
| 20374209 |
| 20374445 |
| 20374446 |
| 20374447 |
| 20378600 |
| 20381876 |
| 20382001 |
| 20388566 |
| 20394313 |
| 20394314 |
| 20394315 |
| 20394316 |
| 20394318 |
| 20394319 |
| 20394320 |
| 20394321 |
| 20394323 |
| 20394324 |
| 20394325 |
| 20395556 |
| 20398302 |
| 20400291 |
| 20401617 |
| 20406032 |
| 20406316 |
| 20406508 |
| 20408922 |
| 20408923 |
| 20408926 |
| 20408927 |
| 20408928 |
| 20408929 |
| 20408930 |
| 20408933 |
| 20408942 |
| 20411686 |
| 20411688 |
| 20411690 |
| 20411691 |
| 20411692 |
| 20411693 |
| 20411695 |
| 20411696 |
| 20411697 |
| 20419135 |
| 20421201 |
| 20421251 |
| 20423584 |
| 20423926 |
| 20424352 |
| 20427615 |
| 20430605 |
| 20437313 |
| 20438143 |
| 20438325 |
| 20439200 |
| 20439796 |
| 20441593 |
| 20444853 |
| 20449805 |
| 20450941 |
| 20453022 |
| 20454497 |
| 20456661 |
| 20457689 |
| 20465015 |
| 20465016 |
| 20465017 |
| 20465018 |
| 20465019 |
| 20465021 |
| 20466584 |
| 20466675 |
| 20469801 |
| 20469802 |
| 20469803 |
| 20469806 |
| 20469807 |
| 20469808 |
| 20469809 |
| 20469810 |
| 20469812 |
| 20469813 |
| 20469814 |
| 20470416 |
| 20470906 |
| 20471106 |
| 20473719 |
| 20477951 |
| 20477952 |
| 20477953 |
| 20477954 |
| 20478849 |
| 20479040 |
| 20479232 |
| 20479950 |
| 20485528 |
| 20488856 |
| 20489690 |
| 20495105 |
| 20498222 |
| 20498441 |
| 20507626 |
| 20508215 |
| 20508218 |
| 20508951 |
| 20510182 |
| 20513034 |
| 20519376 |
| 20523303 |
| 20525714 |
| 20534826 |
| 20537611 |
| 20538960 |
| 20541968 |
| 20542742 |
| 20544939 |
| 20545482 |
| 20545836 |
| 20545837 |
| 20545838 |
| 20545839 |
| 20546581 |
| 20550013 |
| 20550016 |
| 20550017 |
| 20550020 |
| 20550021 |
| 20550022 |
| 20550023 |
| 20550024 |
| 20550026 |
| 20551846 |
| 20559476 |
| 20561172 |
| 20561173 |
| 20561174 |
| 20561176 |
| 20561177 |
| 20561178 |
| 20561179 |
| 20561180 |
| 20562120 |
| 20566740 |
| 20570124 |
| 20570482 |
| 20572413 |
| 20572415 |
| 20572416 |
| 20572417 |
| 20572419 |
| 20572420 |
| 20572423 |
| 20576847 |
| 20585000 |
| 20587858 |
| 20595146 |
| 20595158 |
| 20596541 |
| 20598584 |
| 20604637 |
| 20614854 |
| 20614855 |
| 20614857 |
| 20614858 |
| 20614859 |
| 20615932 |
| 20620078 |
| 20620805 |
| 20624840 |
| 20625114 |
| 20626046 |
| 20626611 |
| 20626613 |
| 20628509 |
| 20628643 |
| 20629937 |
| 20629938 |
| 20629939 |
| 20630707 |
| 20634711 |
| 20637689 |
| 20639438 |
| 20650989 |
| 20651279 |
| 20653452 |
| 20656013 |
| 20656942 |
| 20668263 |
| 20668283 |
| 20668840 |
| 20668925 |
| 20670413 |
| 20673289 |
| 20673290 |
| 20673291 |
| 20673292 |
| 20673648 |
| 20682605 |
| 20682669 |
| 20683768 |
| 20685163 |
| 20685829 |
| 20688812 |
| 20689107 |
| 20699599 |
| 20701789 |
| 20702607 |
| 20704644 |
| 20704645 |
| 20705231 |
| 20708004 |
| 20708275 |
| 20711453 |
| 20712854 |
| 20715166 |
| 20719289 |
| 20719433 |
| 20722819 |
| 20723022 |
| 20723232 |
| 20723551 |
| 20723785 |
| 20724559 |
| 20724569 |
| 20724769 |
| 20725134 |
| 20726278 |
| 20726279 |
| 20726280 |
| 20726281 |
| 20726282 |
| 20726283 |
| 20726285 |
| 20727066 |
| 20729099 |
| 20731498 |
| 20732629 |
| 20739559 |
| 20795887 |
| 20808975 |
| 20809027 |
| 20810449 |
| 20813580 |
| 20814340 |
| 20815182 |
| 20815183 |
| 20815184 |
| 20815188 |
| 20815189 |
| 20815190 |
| 20815191 |
| 20815194 |
| 20815195 |
| 20815898 |
| 20817553 |
| 20817710 |
| 20817711 |
| 20819145 |
| 20819146 |
| 20823756 |
| 20825464 |
| 20829636 |
| 20844115 |
| 20846669 |
| 20846992 |
| 20847078 |
| 20850067 |
| 20850231 |
| 20857859 |
| 20857861 |
| 20857862 |
| 20857863 |
| 20857864 |
| 20857865 |
| 20857866 |
| 20857867 |
| 20857869 |
| 20857873 |
| 20860564 |
| 20870457 |
| 20878849 |
| 20880101 |
| 20887393 |
| 20887394 |
| 20887395 |
| 20887396 |
| 20888921 |
| 20889165 |
| 20890980 |
| 20920152 |
| 20920190 |
| 20921542 |
| 20924835 |
| 20929465 |
| 20935105 |
| 20936662 |
| 20946334 |
| 20946437 |
| 20946684 |
| 20946745 |
| 20948522 |
| 20955865 |
| 20955866 |
| 20955867 |
| 20956420 |
| 20957841 |
| 20957843 |
| 20957844 |
| 20957845 |
| 20957846 |
| 20957847 |
| 20957849 |
| 20957850 |
| 20959329 |
| 20962287 |
| 20966412 |
| 20966622 |
| 20970088 |
| 20974775 |
| 20975052 |
| 20977513 |
| 20978224 |
| 20978444 |
| 20980591 |
| 21029511 |
| 21037995 |
| 21040938 |
| 21041596 |
| 21047418 |
| 21058849 |
| 21058868 |
| 21059713 |
| 21060117 |
| 21061852 |
| 21061854 |
| 21061856 |
| 21061857 |
| 21061859 |
| 21061861 |
| 21070424 |
| 21074577 |
| 21075318 |
| 21075771 |
| 21076087 |
| 21083682 |
| 21087712 |
| 21093364 |
| 21093372 |
| 21095200 |
| 21097658 |
| 21098528 |
| 21102986 |
| 21102989 |
| 21102990 |
| 21102992 |
| 21102997 |
| 21102998 |
| 21105175 |
| 21106625 |
| 21116652 |
| 21118712 |
| 21118908 |
| 21120123 |
| 21120124 |
| 21120125 |
| 21120126 |
| 21120127 |
| 21120130 |
| 21120131 |
| 21120152 |
| 21122092 |
| 21123763 |
| 21129054 |
| 21129351 |
| 21130696 |
| 21132554 |
| 21135162 |
| 21144676 |
| 21144798 |
| 21145786 |
| 21146189 |
| 21146465 |
| 21151998 |
| 21152066 |
| 21156746 |
| 21161317 |
| 21163493 |
| 21165351 |
| 21165665 |
| 21168395 |
| 21169344 |
| 21170381 |
| 21171765 |
| 21175013 |
| 21177837 |
| 21177842 |
| 21178589 |
| 21183327 |
| 21183328 |
| 21188675 |
| 21188850 |
| 21190045 |
| 21193534 |
| 21194997 |
| 21199039 |
| 21203365 |
| 21203367 |
| 21203368 |
| 21203376 |
| 21203377 |
| 21206543 |
| 21206544 |
| 21206546 |
| 21206547 |
| 21206548 |
| 21206549 |
| 21206743 |
| 21206744 |
| 21218289 |
| 21220260 |
| 21220756 |
| 21232930 |
| 21233171 |
| 21233264 |
| 21237441 |
| 21237517 |
| 21242462 |
| 21243280 |
| 21243655 |
| 21247540 |
| 21252646 |
| 21255141 |
| 21256770 |
| 21256805 |
| 21257181 |
| 21261767 |
| 21262888 |
| 21263013 |
| 21265248 |
| 21269278 |
| 21273994 |
| 21277182 |
| 21277183 |
| 21278167 |
| 21278698 |
| 21281369 |
| 21281471 |
| 21282549 |
| 21283819 |
| 21286230 |
| 21286243 |
| 21286244 |
| 21286245 |
| 21286250 |
| 21286251 |
| 21286252 |
| 21286495 |
| 21286497 |
| 21286498 |
| 21289163 |
| 21292329 |
| 21294800 |
| 21296508 |
| 21302271 |
| 21303422 |
| 21303806 |
| 21310653 |
| 21310729 |
| 21312040 |
| 21314643 |
| 21314649 |
| 21316267 |
| 21316300 |
| 21317406 |
| 21320949 |
| 21322021 |
| 21323679 |
| 21324013 |
| 21324900 |
| 21324979 |
| 21325523 |
| 21330338 |
| 21330468 |
| 21334465 |
| 21334973 |
| 21340034 |
| 21340475 |
| 21344040 |
| 21344046 |
| 21344047 |
| 21344048 |
| 21344049 |
| 21344051 |
| 21344054 |
| 21344056 |
| 21349763 |
| 21349927 |
| 21350844 |
| 21352389 |
| 21353642 |
| 21354892 |
| 21357280 |
| 21357341 |
| 21357344 |
| 21358601 |
| 21358603 |
| 21358842 |
| 21358845 |
| 21358846 |
| 21358847 |
| 21358848 |
| 21358849 |
| 21358850 |
| 21358853 |
| 21358855 |
| 21359089 |
| 21360253 |
| 21372306 |
| 21381949 |
| 21383212 |
| 21383213 |
| 21383214 |
| 21383215 |
| 21385420 |
| 21386998 |
| 21388906 |
| 21389226 |
| 21390907 |
| 21392474 |
| 21393396 |
| 21397252 |
| 21397626 |
| 21399726 |
| 21402472 |
| 21403097 |
| 21406511 |
| 21408616 |
| 21410809 |
| 21411507 |
| 21414566 |
| 21415034 |
| 21415310 |
| 21415314 |
| 21415315 |
| 21420904 |
| 21429282 |
| 21430147 |
| 21432824 |
| 21434971 |
| 21435064 |
| 21435231 |
| 21436459 |
| 21437285 |
| 21441128 |
| 21443301 |
| 21447050 |
| 21448129 |
| 21450184 |
| 21452251 |
| 21454042 |
| 21459838 |
| 21461321 |
| 21461323 |
| 21461324 |
| 21461325 |
| 21461326 |
| 21461328 |
| 21461329 |
| 21461330 |
| 21461332 |
| 21463967 |
| 21464115 |
| 21465680 |
| 21467248 |
| 21471283 |
| 21474924 |
| 21474933 |
| 21475378 |
| 21477910 |
| 21480159 |
| 21482029 |
| 21482065 |
| 21482604 |
| 21484777 |
| 21489004 |
| 21489869 |
| 21491232 |
| 21496357 |
| 21505779 |
| 21507144 |
| 21508507 |
| 21509329 |
| 21509330 |
| 21509331 |
| 21509332 |
| 21509334 |
| 21509335 |
| 21509337 |
| 21513820 |
| 21515199 |
| 21517173 |
| 21518065 |
| 21518066 |
| 21522045 |
| 21522061 |
| 21523471 |
| 21523492 |
| 21525277 |
| 21527006 |
| 21532949 |
| 21532950 |
| 21532951 |
| 21532954 |
| 21532957 |
| 21532958 |
| 21532961 |
| 21535281 |
| 21537164 |
| 21537196 |
| 21538678 |
| 21543596 |
| 21544202 |
| 21546480 |
| 21553979 |
| 21554464 |
| 21555678 |
| 21555717 |
| 21557231 |
| 21557740 |
| 21561903 |
| 21564364 |
| 21567239 |
| 21569264 |
| 21570250 |
| 21570347 |
| 21572244 |
| 21573904 |
| 21575815 |
| 21590342 |
| 21596794 |
| 21601293 |
| 21605390 |
| 21605479 |
| 21608086 |
| 21614574 |
| 21615580 |
| 21620678 |
| 21622518 |
| 21622583 |
| 21622590 |
| 21622591 |
| 21623597 |
| 21626120 |
| 21627765 |
| 21629357 |
| 21629358 |
| 21629359 |
| 21629360 |
| 21629362 |
| 21629366 |
| 21629368 |
| 21632666 |
| 21632713 |
| 21633397 |
| 21636668 |
| 21638116 |
| 21645911 |
| 21646006 |
| 21652261 |
| 21655926 |
| 21656087 |
| 21664182 |
| 21664380 |
| 21668552 |
| 21668806 |
| 21669584 |
| 21672069 |
| 21673002 |
| 21676224 |
| 21677161 |
| 21677894 |
| 21677895 |
| 21677896 |
| 21677898 |
| 21677901 |
| 21681797 |
| 21683450 |
| 21684219 |
| 21688915 |
| 21689982 |
| 21690336 |
| 21691915 |
| 21695061 |
| 21696423 |
| 21701911 |
| 21702865 |
| 21704134 |
| 21704557 |
| 21707809 |
| 21708802 |
| 21708803 |
| 21709609 |
| 21709634 |
| 21715230 |
| 21715510 |
| 21719490 |
| 21720936 |
| 21722013 |
| 21724373 |
| 21731137 |
| 21731138 |
| 21731139 |
| 21731140 |
| 21731141 |
| 21731143 |
| 21731144 |
| 21731145 |
| 21733182 |
| 21734253 |
| 21742478 |
| 21743341 |
| 21745579 |
| 21748445 |
| 21752133 |
| 21752673 |
| 21753010 |
| 21760489 |
| 21762445 |
| 21762553 |
| 21762556 |
| 21762560 |
| 21764369 |
| 21766076 |
| 21770763 |
| 21771748 |
| 21775199 |
| 21784661 |
| 21785370 |
| 21785373 |
| 21785899 |
| 21789214 |
| 21795377 |
| 21795436 |
| 21795758 |
| 21796355 |
| 21797835 |
| 21798674 |
| 21799124 |
| 21799479 |
| 21799746 |
| 21799880 |
| 21804661 |
| 21804663 |
| 21804664 |
| 21804665 |
| 21804666 |
| 21804667 |
| 21804669 |
| 21804676 |
| 21806564 |
| 21808447 |
| 21815957 |
| 21816697 |
| 21819338 |
| 21820299 |
| 21825266 |
| 21827556 |
| 21828324 |
| 21829643 |
| 21836106 |
| 21836132 |
| 21842957 |
| 21842997 |
| 21843282 |
| 21846365 |
| 21848707 |
| 21851192 |
| 21851924 |
| 21852330 |
| 21852408 |
| 21853136 |
| 21853548 |
| 21854969 |
| 21856678 |
| 21857929 |
| 21859438 |
| 21859921 |
| 21859975 |
| 21864519 |
| 21866046 |
| 21871836 |
| 21872458 |
| 21873265 |
| 21873695 |
| 21873945 |
| 21875813 |
| 21876072 |
| 21878574 |
| 21880196 |
| 21884159 |
| 21884956 |
| 21885389 |
| 21885490 |
| 21885593 |
| 21886354 |
| 21886355 |
| 21886356 |
| 21886357 |
| 21886358 |
| 21886359 |
| 21886360 |
| 21886361 |
| 21886362 |
| 21886770 |
| 21886801 |
| 21888458 |
| 21889324 |
| 21889359 |
| 21890825 |
| 21895822 |
| 21897768 |
| 21897770 |
| 21897771 |
| 21897772 |
| 21897774 |
| 21897775 |
| 21897776 |
| 21903027 |
| 21903736 |
| 21906021 |
| 21911350 |
| 21915482 |
| 21917047 |
| 21924664 |
| 21925567 |
| 21925945 |
| 21926292 |
| 21928913 |
| 21932189 |
| 21932336 |
| 21933909 |
| 21934125 |
| 21935265 |
| 21940204 |
| 21940205 |
| 21942668 |
| 21943153 |
| 21948205 |
| 21949422 |
| 21950736 |
| 21952668 |
| 21953381 |
| 21955170 |
| 21955220 |
| 21961447 |
| 21965189 |
| 21965227 |
| 21966060 |
| 21966061 |
| 21966062 |
| 21966066 |
| 21966069 |
| 21966070 |
| 21966072 |
| 21966073 |
| 21966074 |
| 21966075 |
| 21967121 |
| 21967958 |
| 21968750 |
| 21969928 |
| 21977863 |
| 21986193 |
| 21988087 |
| 21988108 |
| 21988168 |
| 21988551 |
| 21990077 |
| 21996663 |
| 21996665 |
| 21999153 |
| 22000536 |
| 22003337 |
| 22003340 |
| 22003341 |
| 22003342 |
| 22003344 |
| 22003346 |
| 22003347 |
| 22003348 |
| 22003980 |
| 22004346 |
| 22004376 |
| 22004426 |
| 22009579 |
| 22010780 |
| 22012805 |
| 22016445 |
| 22016588 |
| 22017727 |
| 22023164 |
| 22025580 |
| 22029569 |
| 22030801 |
| 22032708 |
| 22033120 |
| 22036598 |
| 22038951 |
| 22039079 |
| 22043118 |
| 22043119 |
| 22043120 |
| 22043121 |
| 22043123 |
| 22043126 |
| 22043127 |
| 22043129 |
| 22043284 |
| 22051093 |
| 22051577 |
| 22058175 |
| 22071385 |
| 22074234 |
| 22075478 |
| 22075482 |
| 22076308 |
| 22077067 |
| 22078131 |
| 22080455 |
| 22080736 |
| 22080786 |
| 22081440 |
| 22084833 |
| 22086518 |
| 22097901 |
| 22102290 |
| 22102513 |
| 22112049 |
| 22114672 |
| 22114954 |
| 22116514 |
| 22118386 |
| 22123526 |
| 22123867 |
| 22123869 |
| 22126225 |
| 22127514 |
| 22129355 |
| 22130149 |
| 22131599 |
| 22131603 |
| 22131604 |
| 22131605 |
| 22131606 |
| 22131607 |
| 22131608 |
| 22131610 |
| 22135343 |
| 22137667 |
| 22140557 |
| 22149126 |
| 22150963 |
| 22151014 |
| 22151079 |
| 22151802 |
| 22152279 |
| 22152403 |
| 22153777 |
| 22156693 |
| 22159098 |
| 22160300 |
| 22162648 |
| 22163330 |
| 22168642 |
| 22171143 |
| 22171197 |
| 22171198 |
| 22171199 |
| 22171200 |
| 22171203 |
| 22171206 |
| 22172966 |
| 22177341 |
| 22182309 |
| 22188071 |
| 22188114 |
| 22188424 |
| 22194182 |
| 22197048 |
| 22199130 |
| 22207114 |
| 22208861 |
| 22210572 |
| 22211627 |
| 22211671 |
| 22214254 |
| 22215376 |
| 22215917 |
| 22215918 |
| 22215919 |
| 22215920 |
| 22215921 |
| 22215922 |
| 22215923 |
| 22215925 |
| 22215928 |
| 22216231 |
| 22217111 |
| 22217496 |
| 22218631 |
| 22218671 |
| 22218837 |
| 22219335 |
| 22221152 |
| 22221543 |
| 22237311 |
| 22239924 |
| 22241742 |
| 22241870 |
| 22244090 |
| 22248509 |
| 22250775 |
| 22250779 |
| 22251024 |
| 22253047 |
| 22254601 |
| 22256131 |
| 22261833 |
| 22262901 |
| 22264851 |
| 22266135 |
| 22267420 |
| 22267762 |
| 22269619 |
| 22273428 |
| 22275251 |
| 22275538 |
| 22275917 |
| 22277375 |
| 22281448 |
| 22282051 |
| 22282488 |
| 22286779 |
| 22292580 |
| 22294807 |
| 22294809 |
| 22294812 |
| 22294814 |
| 22294815 |
| 22294816 |
| 22294818 |
| 22294820 |
| 22295122 |
| 22298286 |
| 22300710 |
| 22303998 |
| 22307639 |
| 22309157 |
| 22309313 |
| 22309485 |
| 22309720 |
| 22310840 |
| 22312420 |
| 22313797 |
| 22317892 |
| 22320933 |
| 22323303 |
| 22326304 |
| 22326831 |
| 22326832 |
| 22327621 |
| 22328924 |
| 22329363 |
| 22329989 |
| 22331514 |
| 22331832 |
| 22333177 |
| 22334803 |
| 22334806 |
| 22334810 |
| 22334811 |
| 22334812 |
| 22334813 |
| 22334814 |
| 22334815 |
| 22337162 |
| 22340304 |
| 22340757 |
| 22341191 |
| 22341378 |
| 22342365 |
| 22343331 |
| 22344942 |
| 22355644 |
| 22356670 |
| 22357064 |
| 22357722 |
| 22362339 |
| 22362853 |
| 22363414 |
| 22363484 |
| 22364822 |
| 22366695 |
| 22367137 |
| 22367162 |
| 22367233 |
| 22377572 |
| 22377800 |
| 22379238 |
| 22379239 |
| 22379241 |
| 22379246 |
| 22379248 |
| 22381437 |
| 22383478 |
| 22388231 |
| 22390611 |
| 22392181 |
| 22396174 |
| 22401881 |
| 22402419 |
| 22404078 |
| 22404768 |
| 22405221 |
| 22406004 |
| 22406033 |
| 22407477 |
| 22408018 |
| 22408210 |
| 22414938 |
| 22415141 |
| 22415568 |
| 22417163 |
| 22417386 |
| 22420746 |
| 22424053 |
| 22426500 |
| 22427104 |
| 22428562 |
| 22429599 |
| 22429651 |
| 22430527 |
| 22431801 |
| 22433978 |
| 22434739 |
| 22438469 |
| 22438995 |
| 22440550 |
| 22440587 |
| 22441739 |
| 22441744 |
| 22445231 |
| 22445783 |
| 22450224 |
| 22451332 |
| 22451703 |
| 22451917 |
| 22451933 |
| 22455464 |
| 22457736 |
| 22459776 |
| 22461045 |
| 22461134 |
| 22463493 |
| 22463694 |
| 22465451 |
| 22467982 |
| 22467983 |
| 22467984 |
| 22467985 |
| 22467986 |
| 22467987 |
| 22467988 |
| 22467989 |
| 22467990 |
| 22467991 |
| 22467992 |
| 22467994 |
| 22468689 |
| 22470524 |
| 22471712 |
| 22472633 |
| 22481243 |
| 22482846 |
| 22486223 |
| 22494030 |
| 22496545 |
| 22496749 |
| 22503380 |
| 22505856 |
| 22505857 |
| 22505858 |
| 22505860 |
| 22505861 |
| 22513492 |
| 22515860 |
| 22516529 |
| 22521226 |
| 22522445 |
| 22523181 |
| 22523356 |
| 22523363 |
| 22523904 |
| 22525467 |
| 22525967 |
| 22529208 |
| 22530064 |
| 22531104 |
| 22537466 |
| 22538736 |
| 22539169 |
| 22539566 |
| 22539569 |
| 22542787 |
| 22543257 |
| 22545062 |
| 22545912 |
| 22547885 |
| 22547887 |
| 22547890 |
| 22547891 |
| 22547893 |
| 22547894 |
| 22547895 |
| 22547896 |
| 22549286 |
| 22551658 |
| 22552256 |
| 22553114 |
| 22554778 |
| 22556023 |
| 22561644 |
| 22562077 |
| 22562273 |
| 22564039 |
| 22570363 |
| 22572632 |
| 22573693 |
| 22578824 |
| 22578889 |
| 22580862 |
| 22581485 |
| 22585775 |
| 22591117 |
| 22592574 |
| 22593062 |
| 22594455 |
| 22596043 |
| 22609024 |
| 22610391 |
| 22612581 |
| 22617164 |
| 22622709 |
| 22623888 |
| 22624036 |
| 22624953 |
| 22633476 |
| 22633696 |
| 22643156 |
| 22644618 |
| 22645449 |
| 22647496 |
| 22651897 |
| 22653715 |
| 22654197 |
| 22654198 |
| 22654203 |
| 22655109 |
| 22658601 |
| 22659030 |
| 22659474 |
| 22666142 |
| 22666278 |
| 22669298 |
| 22669471 |
| 22669593 |
| 22671994 |
| 22676966 |
| 22677350 |
| 22679060 |
| 22688546 |
| 22690985 |
| 22691500 |
| 22693128 |
| 22700841 |
| 22700842 |
| 22701379 |
| 22701381 |
| 22701382 |
| 22701383 |
| 22701388 |
| 22701389 |
| 22701390 |
| 22702839 |
| 22705247 |
| 22705609 |
| 22708533 |
| 22709334 |
| 22709822 |
| 22710148 |
| 22715089 |
| 22716195 |
| 22723291 |
| 22724534 |
| 22727156 |
| 22727630 |
| 22727943 |
| 22729987 |
| 22738694 |
| 22738735 |
| 22742082 |
| 22742416 |
| 22742436 |
| 22743670 |
| 22746298 |
| 22747735 |
| 22748486 |
| 22748491 |
| 22749755 |
| 22750520 |
| 22751369 |
| 22752291 |
| 22752878 |
| 22754038 |
| 22754039 |
| 22754040 |
| 22754042 |
| 22754043 |
| 22754044 |
| 22754045 |
| 22754046 |
| 22754047 |
| 22757951 |
| 22758620 |
| 22760574 |
| 22760893 |
| 22761257 |
| 22761732 |
| 22761881 |
| 22762354 |
| 22763233 |
| 22768272 |
| 22770599 |
| 22770684 |
| 22770698 |
| 22771125 |
| 22773271 |
| 22780916 |
| 22790918 |
| 22791640 |
| 22792235 |
| 22795049 |
| 22795497 |
| 22796931 |
| 22807627 |
| 22808287 |
| 22815387 |
| 22815388 |
| 22818237 |
| 22821607 |
| 22822640 |
| 22823997 |
| 22824065 |
| 22831887 |
| 22832325 |
| 22832540 |
| 22835074 |
| 22835145 |
| 22835611 |
| 22836655 |
| 22837368 |
| 22837377 |
| 22839359 |
| 22841028 |
| 22841032 |
| 22844441 |
| 22845651 |
| 22845675 |
| 22846722 |
| 22848006 |
| 22850349 |
| 22850546 |
| 22851802 |
| 22851803 |
| 22851804 |
| 22851805 |
| 22851806 |
| 22851807 |
| 22851808 |
| 22851811 |
| 22851812 |
| 22851813 |
| 22853649 |
| 22854768 |
| 22858121 |
| 22858167 |
| 22858235 |
| 22862846 |
| 22862979 |
| 22863161 |
| 22864597 |
| 22866540 |
| 22869826 |
| 22869829 |
| 22869830 |
| 22871062 |
| 22871531 |
| 22871637 |
| 22877997 |
| 22879917 |
| 22881342 |
| 22883600 |
| 22884306 |
| 22884414 |
| 22888782 |
| 22889464 |
| 22890070 |
| 22891194 |
| 22892811 |
| 22893765 |
| 22893766 |
| 22893767 |
| 22893768 |
| 22893769 |
| 22893770 |
| 22893773 |
| 22893774 |
| 22893775 |
| 22895593 |
| 22898005 |
| 22901794 |
| 22902291 |
| 22903961 |
| 22903967 |
| 22906153 |
| 22906794 |
| 22907117 |
| 22908930 |
| 22910247 |
| 22910327 |
| 22912423 |
| 22912833 |
| 22916259 |
| 22917967 |
| 22921179 |
| 22921588 |
| 22924964 |
| 22926090 |
| 22935396 |
| 22935746 |
| 22938169 |
| 22938861 |
| 22939103 |
| 22942500 |
| 22942502 |
| 22942504 |
| 22942505 |
| 22942507 |
| 22942508 |
| 22945405 |
| 22946733 |
| 22947657 |
| 22951185 |
| 22952630 |
| 22952909 |
| 22959616 |
| 22960270 |
| 22964793 |
| 22966034 |
| 22967281 |
| 22968669 |
| 22972835 |
| 22974470 |
| 22984209 |
| 22985414 |
| 22989072 |
| 22991449 |
| 22993169 |
| 22996794 |
| 23001190 |
| 23005259 |
| 23006602 |
| 23011061 |
| 23018902 |
| 23018907 |
| 23019313 |
| 23023679 |
| 23023681 |
| 23024431 |
| 23024432 |
| 23024433 |
| 23024438 |
| 23024439 |
| 23024440 |
| 23025266 |
| 23025547 |
| 23026127 |
| 23027175 |
| 23027917 |
| 23029352 |
| 23031583 |
| 23032246 |
| 23036264 |
| 23041446 |
| 23043357 |
| 23046065 |
| 23051618 |
| 23051680 |
| 23056581 |
| 23057438 |
| 23061443 |
| 23063043 |
| 23063144 |
| 23063244 |
| 23065547 |
| 23066359 |
| 23066360 |
| 23066361 |
| 23066362 |
| 23066363 |
| 23066364 |
| 23066367 |
| 23066368 |
| 23066370 |
| 23067390 |
| 23070339 |
| 23071214 |
| 23071658 |
| 23072292 |
| 23073072 |
| 23075634 |
| 23082111 |
| 23086669 |
| 23086863 |
| 23088174 |
| 23088719 |
| 23088735 |
| 23090526 |
| 23091013 |
| 23093815 |
| 23107000 |
| 23108384 |
| 23109097 |
| 23110178 |
| 23115214 |
| 23115396 |
| 23115398 |
| 23115399 |
| 23115401 |
| 23115402 |
| 23115405 |
| 23118258 |
| 23119069 |
| 23121320 |
| 23124650 |
| 23127585 |
| 23129488 |
| 23129543 |
| 23136831 |
| 23136848 |
| 23137332 |
| 23137824 |
| 23139361 |
| 23141932 |
| 23144701 |
| 23144920 |
| 23145159 |
| 23145881 |
| 23145885 |
| 23148451 |
| 23149024 |
| 23150286 |
| 23154449 |
| 23155414 |
| 23159421 |
| 23162043 |
| 23166147 |
| 23166808 |
| 23167935 |
| 23171222 |
| 23171248 |
| 23171375 |
| 23171414 |
| 23171567 |
| 23176607 |
| 23179074 |
| 23179139 |
| 23181913 |
| 23182016 |
| 23185361 |
| 23186084 |
| 23186226 |
| 23186649 |
| 23192600 |
| 23193113 |
| 23193115 |
| 23197238 |
| 23204602 |
| 23204603 |
| 23204604 |
| 23204606 |
| 23204609 |
| 23204610 |
| 23205513 |
| 23205856 |
| 23205895 |
| 23212498 |
| 23216856 |
| 23216995 |
| 23217021 |
| 23217056 |
| 23217092 |
| 23218998 |
| 23219072 |
| 23219144 |
| 23219145 |
| 23220839 |
| 23221728 |
| 23226400 |
| 23226554 |
| 23227139 |
| 23228137 |
| 23228180 |
| 23236418 |
| 23239460 |
| 23243396 |
| 23243397 |
| 23243398 |
| 23243400 |
| 23243401 |
| 23244163 |
| 23244456 |
| 23256572 |
| 23257990 |
| 23265919 |
| 23270533 |
| 23272670 |
| 23278677 |
| 23279292 |
| 23283336 |
| 23284971 |
| 23286303 |
| 23286392 |
| 23287986 |
| 23288434 |
| 23288967 |
| 23288968 |
| 23288970 |
| 23288973 |
| 23288974 |
| 23288977 |
| 23288982 |
| 23294420 |
| 23295586 |
| 23295588 |
| 23297197 |
| 23300931 |
| 23300993 |
| 23306904 |
| 23308333 |
| 23314584 |
| 23315234 |
| 23319532 |
| 23319901 |
| 23319902 |
| 23319903 |
| 23319905 |
| 23319906 |
| 23324334 |
| 23324875 |
| 23326012 |
| 23326598 |
| 23326604 |
| 23328840 |
| 23331266 |
| 23331712 |
| 23331757 |
| 23331844 |
| 23337557 |
| 23339829 |
| 23339991 |
| 23340087 |
| 23340852 |
| 23342100 |
| 23342127 |
| 23345934 |
| 23347467 |
| 23349453 |
| 23350718 |
| 23352028 |
| 23352420 |
| 23354511 |
| 23355343 |
| 23359027 |
| 23360346 |
| 23360632 |
| 23361061 |
| 23363423 |
| 23366374 |
| 23367114 |
| 23372263 |
| 23372265 |
| 23372266 |
| 23372267 |
| 23372268 |
| 23372269 |
| 23372271 |
| 23372273 |
| 23372274 |
| 23372276 |
| 23372461 |
| 23372462 |
| 23372463 |
| 23372466 |
| 23372468 |
| 23372469 |
| 23374054 |
| 23374906 |
| 23375931 |
| 23376698 |
| 23376797 |
| 23378502 |
| 23380170 |
| 23382975 |
| 23383010 |
| 23392199 |
| 23393417 |
| 23394437 |
| 23398021 |
| 23398048 |
| 23398120 |
| 23398166 |
| 23398203 |
| 23398262 |
| 23402597 |
| 23403781 |
| 23406753 |
| 23409055 |
| 23409736 |
| 23412355 |
| 23412676 |
| 23412897 |
| 23414228 |
| 23418478 |
| 23418586 |
| 23419057 |
| 23420264 |
| 23421840 |
| 23425048 |
| 23425321 |
| 23426232 |
| 23426660 |
| 23428257 |
| 23428653 |
| 23429871 |
| 23431305 |
| 23431309 |
| 23432361 |
| 23432533 |
| 23436793 |
| 23437852 |
| 23438443 |
| 23440187 |
| 23445081 |
| 23447518 |
| 23449493 |
| 23449753 |
| 23449886 |
| 23453905 |
| 23457611 |
| 23459152 |
| 23460685 |
| 23466223 |
| 23467365 |
| 23471157 |
| 23471270 |
| 23473922 |
| 23474058 |
| 23476674 |
| 23479184 |
| 23479616 |
| 23481119 |
| 23483378 |
| 23483886 |
| 23489400 |
| 23493388 |
| 23493528 |
| 23493642 |
| 23493815 |
| 23493839 |
| 23493949 |
| 23495953 |
| 23497046 |
| 23497827 |
| 23497828 |
| 23498846 |
| 23499514 |
| 23503808 |
| 23504639 |
| 23504939 |
| 23505171 |
| 23507270 |
| 23507553 |
| 23509471 |
| 23509903 |
| 23509952 |
| 23509990 |
| 23512653 |
| 23515262 |
| 23516146 |
| 23520548 |
| 23521019 |
| 23522093 |
| 23523432 |
| 23524009 |
| 23524039 |
| 23525306 |
| 23527213 |
| 23531686 |
| 23535165 |
| 23538032 |
| 23538745 |
| 23543909 |
| 23544134 |
| 23544995 |
| 23551728 |
| 23551934 |
| 23552445 |
| 23554978 |
| 23555220 |
| 23557073 |
| 23558371 |
| 23560045 |
| 23560353 |
| 23560612 |
| 23562184 |
| 23564028 |
| 23564997 |
| 23564998 |
| 23565000 |
| 23565001 |
| 23565004 |
| 23565005 |
| 23565006 |
| 23565669 |
| 23567762 |
| 23568000 |
| 23568594 |
| 23569090 |
| 23571526 |
| 23575863 |
| 23577218 |
| 23578347 |
| 23583623 |
| 23585743 |
| 23585745 |
| 23585747 |
| 23585749 |
| 23585750 |
| 23585752 |
| 23585753 |
| 23589584 |
| 23589814 |
| 23591911 |
| 23592753 |
| 23593990 |
| 23599393 |
| 23600534 |
| 23601161 |
| 23604499 |
| 23609427 |
| 23611716 |
| 23613841 |
| 23618484 |
| 23620639 |
| 23622883 |
| 23623452 |
| 23623910 |
| 23623949 |
| 23624560 |
| 23628083 |
| 23633116 |
| 23633746 |
| 23633747 |
| 23633748 |
| 23633749 |
| 23633750 |
| 23633753 |
| 23633754 |
| 23633755 |
| 23633759 |
| 23633760 |
| 23633761 |
| 23637139 |
| 23637744 |
| 23637783 |
| 23642544 |
| 23645550 |
| 23649748 |
| 23650511 |
| 23651054 |
| 23652374 |
| 23658732 |
| 23661764 |
| 23663216 |
| 23666635 |
| 23669108 |
| 23670279 |
| 23671601 |
| 23674932 |
| 23674933 |
| 23674935 |
| 23674938 |
| 23674939 |
| 23674941 |
| 23675433 |
| 23681944 |
| 23682620 |
| 23682639 |
| 23690272 |
| 23690360 |
| 23690688 |
| 23691095 |
| 23692173 |
| 23692581 |
| 23695990 |
| 23697706 |
| 23699749 |
| 23701424 |
| 23706838 |
| 23709430 |
| 23711813 |
| 23713101 |
| 23714281 |
| 23715755 |
| 23720185 |
| 23720415 |
| 23721582 |
| 23722610 |
| 23723696 |
| 23728906 |
| 23729925 |
| 23729927 |
| 23729928 |
| 23729929 |
| 23729930 |
| 23729931 |
| 23729932 |
| 23729933 |
| 23729934 |
| 23729935 |
| 23729936 |
| 23729937 |
| 23729938 |
| 23729939 |
| 23729940 |
| 23731062 |
| 23731205 |
| 23731773 |
| 23731915 |
| 23732344 |
| 23733938 |
| 23734235 |
| 23734252 |
| 23735187 |
| 23736744 |
| 23738015 |
| 23739529 |
| 23744568 |
| 23745721 |
| 23746724 |
| 23746737 |
| 23750232 |
| 23751915 |
| 23752209 |
| 23755173 |
| 23756334 |
| 23759408 |
| 23763708 |
| 23764006 |
| 23764862 |
| 23766689 |
| 23768539 |
| 23768840 |
| 23770166 |
| 23770411 |
| 23772184 |
| 23772188 |
| 23772189 |
| 23772191 |
| 23772192 |
| 23772193 |
| 23772195 |
| 23777510 |
| 23778335 |
| 23788221 |
| 23790010 |
| 23797865 |
| 23797955 |
| 23800096 |
| 23800204 |
| 23800287 |
| 23801012 |
| 23801736 |
| 23801738 |
| 23801835 |
| 23803894 |
| 23804084 |
| 23804432 |
| 23805978 |
| 23810359 |
| 23810721 |
| 23811325 |
| 23811525 |
| 23815623 |
| 23821728 |
| 23826169 |
| 23831496 |
| 23831547 |
| 23834036 |
| 23834705 |
| 23835691 |
| 23837871 |
| 23846792 |
| 23846968 |
| 23849514 |
| 23850195 |
| 23850225 |
| 23850226 |
| 23853558 |
| 23853560 |
| 23853561 |
| 23853563 |
| 23853564 |
| 23853565 |
| 23853566 |
| 23860262 |
| 23862291 |
| 23863955 |
| 23864582 |
| 23871259 |
| 23874738 |
| 23880026 |
| 23886318 |
| 23886347 |
| 23887151 |
| 23889686 |
| 23889723 |
| 23891110 |
| 23894293 |
| 23899423 |
| 23902570 |
| 23902787 |
| 23904673 |
| 23904674 |
| 23904675 |
| 23904676 |
| 23904677 |
| 23904679 |
| 23904680 |
| 23904681 |
| 23904682 |
| 23907080 |
| 23907090 |
| 23908148 |
| 23917661 |
| 23917665 |
| 23918740 |
| 23919462 |
| 23919583 |
| 23922121 |
| 23922472 |
| 23923024 |
| 23923994 |
| 23932141 |
| 23936014 |
| 23936084 |
| 23936363 |
| 23937311 |
| 23940231 |
| 23940415 |
| 23940727 |
| 23941878 |
| 23945417 |
| 23946301 |
| 23946702 |
| 23946704 |
| 23946705 |
| 23946706 |
| 23946707 |
| 23946708 |
| 23946709 |
| 23946711 |
| 23946712 |
| 23946713 |
| 23947422 |
| 23950583 |
| 23951064 |
| 23951321 |
| 23951335 |
| 23952346 |
| 23953126 |
| 23954406 |
| 23959732 |
| 23966709 |
| 23967186 |
| 23973762 |
| 23977057 |
| 23979778 |
| 23986109 |
| 23986208 |
| 23987571 |
| 23989018 |
| 23991135 |
| 23993773 |
| 23995177 |
| 23997360 |
| 23997361 |
| 23997364 |
| 23997365 |
| 23997367 |
| 23997369 |
| 23997370 |
| 23997371 |
| 23997695 |
| 23997696 |
| 23997698 |
| 23997700 |
| 23997702 |
| 23997704 |
| 23997705 |
| 23997706 |
| 23998381 |
| 24001143 |
| 24003146 |
| 24005775 |
| 24008357 |
| 24010499 |
| 24010811 |
| 24012066 |
| 24015039 |
| 24015045 |
| 24015253 |
| 24015304 |
| 24018914 |
| 24020811 |
| 24023959 |
| 24025612 |
| 24026624 |
| 24027260 |
| 24027272 |
| 24028643 |
| 24034352 |
| 24037015 |
| 24038728 |
| 24039808 |
| 24041817 |
| 24043914 |
| 24047533 |
| 24049160 |
| 24054762 |
| 24065843 |
| 24068804 |
| 24070180 |
| 24070217 |
| 24074694 |
| 24076138 |
| 24077772 |
| 24082301 |
| 24082302 |
| 24082303 |
| 24082310 |
| 24082311 |
| 24082312 |
| 24082313 |
| 24082314 |
| 24082316 |
| 24086501 |
| 24088722 |
| 24093585 |
| 24094679 |
| 24098425 |
| 24100177 |
| 24101695 |
| 24102962 |
| 24103561 |
| 24107861 |
| 24112372 |
| 24117003 |
| 24118416 |
| 24124962 |
| 24127143 |
| 24127145 |
| 24127147 |
| 24127148 |
| 24127149 |
| 24127150 |
| 24127153 |
| 24130124 |
| 24132058 |
| 24137529 |
| 24143246 |
| 24145819 |
| 24145859 |
| 24146812 |
| 24146857 |
| 24147132 |
| 24149072 |
| 24156237 |
| 24167552 |
| 24171759 |
| 24172110 |
| 24179289 |
| 24179290 |
| 24179291 |
| 24179295 |
| 24179296 |
| 24179297 |
| 24179298 |
| 24179300 |
| 24179302 |
| 24179303 |
| 24179304 |
| 24179306 |
| 24179309 |
| 24184428 |
| 24186593 |
| 24190680 |
| 24195872 |
| 24196305 |
| 24205289 |
| 24205291 |
| 24205327 |
| 24218598 |
| 24222825 |
| 24235891 |
| 24235892 |
| 24235894 |
| 24235895 |
| 24235896 |
| 24235897 |
| 24235898 |
| 24235899 |
| 24235900 |
| 24235903 |
| 24235907 |
| 24236808 |
| 24237356 |
| 24244614 |
| 24247858 |
| 24249825 |
| 24250276 |
| 24260240 |
| 24260280 |
| 24260331 |
| 24265736 |
| 24267255 |
| 24275628 |
| 24278145 |
| 24282197 |
| 24282215 |
| 24282576 |
| 24282622 |
| 24293753 |
| 24293754 |
| 24293760 |
| 24293762 |
| 24293763 |
| 24293764 |
| 24293765 |
| 24293766 |
| 24293767 |
| 24293768 |
| 24293769 |
| 24293770 |
| 24293771 |
| 24293772 |
| 24293773 |
| 24293774 |
| 24293775 |
| 24304942 |
| 24312494 |
| 24312562 |
| 24330358 |
| 24330387 |
| 24335380 |
| 24340283 |
| 24340285 |
| 24340286 |
| 24340287 |
| 24340291 |
| 24340295 |
| 24349191 |
| 24349251 |
| 24349492 |
| 24349512 |
| 24349654 |
| 24359272 |
| 24367602 |
| 24371836 |
| 24376607 |
| 24377840 |
| 24392615 |
| 24431309 |
| 24431310 |
| 24453895 |
| 24469876 |
